# Glutathione in Skin Aging and Tissue Regeneration: A Systematic Review of Molecular Mechanisms, Redox Modulation, and Biomedical Implications

**DOI:** 10.3390/molecules31060981

**Published:** 2026-03-15

**Authors:** Cristina Stanescu, Iulia Chiscop, Monica Boev, Georgiana Daniela Stanescu, Madalina Nicoleta Matei

**Affiliations:** 1 Department of Morphological and Functional Sciences, Faculty of Medicine and Pharmacy, “Dunărea de Jos” University of Galaţi, Domnească Street No. 47, 800008 Galati, Romania; 2 Clinical Surgical Department, Faculty of Medicine and Pharmacy, “Dunărea de Jos” University of Galaţi, Domnească Street No. 47, 800008 Galati, Romania; 3 Department of Pharmaceutical Sciences, Faculty of Medicine and Pharmacy, “Dunărea de Jos” University of Galaţi, Domnească Street No. 47, 800008 Galati, Romania; 4 Faculty of Medicine, “Grigore T. Popa” University of Medicine and Pharmacy, 16 Universitatii Street, 700115 Iasi, Romania; 5 Department of Dental Medicine, Faculty of Medicine and Pharmacy, “Dunărea de Jos” University of Galaţi, Domnească Street No. 47, 800008 Galati, Romania

**Keywords:** glutathione metabolism, redox homeostasis, skin aging, tissue regeneration, bioavailability

## Abstract

Glutathione (GSH) is a central regulator of redox homeostasis, melanogenesis, and cellular repair, and has gained increasing attention in dermatology for its potential roles in skin brightening, anti-aging, and tissue regeneration. This systematic review evaluated molecular, clinical, and translational evidence of glutathione’s applications and safety across different delivery modalities. The review followed PRISMA guidelines and included studies published between 2000 and 2025. A total of 194 studies met the inclusion criteria, evaluating the effectiveness of glutathione in esthetic dermatology and regenerative medicine. Topical and oral glutathione demonstrated favorable effects on pigmentation, skin brightness, hydration, and oxidative stress markers. Injectable glutathione increases systemic levels rapidly, but is associated with short-lasting effects and potential safety concerns. Glutathione S-transferases facilitate the conjugation of glutathione to electrophilic xenobiotics, thereby protecting proteins and nucleic acids from electrophile-induced damage. Glutathione Peroxidase employs GSH as an electron donor to reduce hydrogen peroxide and lipid hydroperoxides, thus protecting membrane lipids, mitochondrial membranes, and DNA from oxidative damage. Glutathione facilitates the regeneration of other antioxidants, such as vitamin C and vitamin E, through redox cycling. A consistent correlation exists between reduced GSH levels and neuronal dysfunction. Elevated GSH levels enhance cellular resistance to oxidative stress and reduce apoptotic signaling. GSH plays a pivotal role in cutaneous aging and tissue repair through redox regulation, mitochondrial protection, and the modulation of inflammatory and extracellular matrix pathways. To elucidate the clinical significance of glutathione, future research should focus on conducting randomized controlled trials, developing standardized formulations, and performing long-term safety assessments.

## 1. Introduction

Skin aging and impaired tissue regeneration represent significant biomedical challenges with esthetic and clinical implications. As the body’s largest organ, the skin is continuously exposed to environmental stressors such as ultraviolet radiation, pollution, and pathogens, while also reflecting intrinsic aging processes driven by genetic and metabolic factors. These cumulative insults lead to structural and functional deterioration, including epidermal thinning, collagen degradation, reduced elasticity, and delayed wound healing [1,2]. The skin’s ability to repair and maintain homeostasis is further compromised by oxidative stress, mitochondrial dysfunction, and cellular senescence, all of which exacerbate the decline in regenerative capacity. The growing demand for anti-aging interventions and regenerative therapies underscores the need for molecular strategies to restore skin integrity and function [3,4].

Oxidative stress plays a central role in the pathophysiology of skin aging and impaired tissue regeneration. As a primary barrier organ, the skin is continuously exposed to endogenous and exogenous sources of reactive oxygen species (ROS), including ultraviolet radiation, pollution, and metabolic byproducts. Excessive production of reactive oxygen species (ROS) disrupts cellular homeostasis, damages DNA, lipids, and proteins, and accelerates the degradation of extracellular matrix components, including collagen and elastin. Redox balance, maintained by endogenous antioxidant systems including glutathione, superoxide dismutase, and catalase, is essential for preserving skin integrity, modulating inflammatory responses, and supporting cellular repair mechanisms [5,6]. Skin aging is characterized by progressive degradation of the extracellular matrix (ECM), primarily driven by oxidative stress and chronic inflammation. Collagen, the most abundant structural protein in the dermis, undergoes fragmentation due to the upregulation of matrix metalloproteinases (MMPs), particularly MMP-1 and MMP-9, which are activated by reactive oxygen species (ROS) and ultraviolet (UV) exposure. This enzymatic breakdown compromises the dermal integrity, leading to reduced tensile strength, wrinkle formation, and impaired wound healing [7,8]. Glutathione (GSH) is a pivotal molecule in regulating cellular redox processes, playing crucial roles in antioxidant defense, detoxification, and immune modulation. Its distinctive thiol-based chemistry facilitates the direct neutralization of reactive oxygen species (ROS), while its participation in enzymatic pathways, such as those involving glutathione peroxidase and reductase, supports dynamic redox cycling. In skin biology, glutathione influences keratinocyte proliferation, melanogenesis, fibroblast activation, and extracellular matrix remodeling, making it a key modulator of both aging and regenerative processes. Unlike other antioxidants, glutathione is endogenously synthesized and tightly regulated, reflecting its essential role in maintaining cellular homeostasis. Emerging evidence also links glutathione depletion to impaired wound healing, increased oxidative damage, and accelerated skin aging [9,10,11,12]. Given its multifaceted functions and clinical relevance, glutathione is a promising molecular target for therapeutic interventions to promote skin rejuvenation, support tissue repair, and mitigate age-related dermatological conditions.

### 1.1. Objectives and Scope of the Review

This review aims to provide a comprehensive, interdisciplinary synthesis of the current knowledge on the role of glutathione in skin aging and tissue regeneration. By integrating molecular, biochemical, and clinical perspectives, this study explores how glutathione modulates redox balance, cellular repair mechanisms, and cutaneous homeostasis. The scope of this review includes an in-depth analysis of glutathione biosynthesis, antioxidant functions, and its impact on keratinocytes, fibroblasts, and extracellular matrix dynamics. This review explores therapeutic strategies for glutathione delivery, emphasizing their clinical significance in esthetic dermatology and regenerative medicine. It particularly addresses the challenges related to bioavailability, formulation stability, and translational applicability. Through this multidimensional approach, this review seeks to clarify the potential of glutathione as a molecular target for anti-aging interventions and skin repair therapies, identify gaps in current research, and propose directions for future investigation.

### 1.2. Glutathione: Molecular Structure

**Figure 1:**
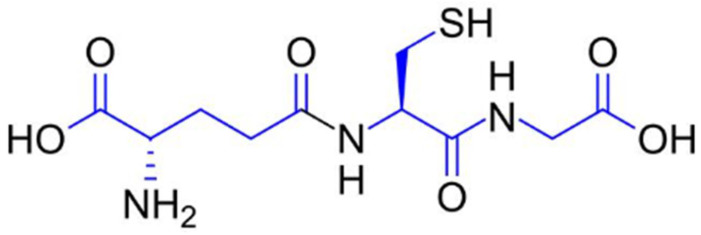
Molecular structure of reduced glutathione (GSH), a tripeptide composed of glutamate, cysteine, and glycine, with the chemical formula C_10_H_17_N_3_O_6_S.

Glutathione (γ-L-glutamyl-L-cysteinyl-glycine) is a low-molecular-weight thiol-tripeptide composed of glutamate, cysteine, and glycine, characterized by a unique γ-peptide bond between the glutamate and cysteine residues (Figure 1). The presence of the cysteinyl thiol (–SH) group confers potent redox activity, enabling glutathione to donate electrons, participate in detoxification reactions, and maintain cellular redox homeostasis. This structural configuration underlies its ability to neutralize reactive oxygen species, regenerate other antioxidants, and modulate melanogenesis and extracellular matrix stability [13,14].

### 1.3. Biosynthesis, Recycling (GSH/GSSG), and Cellular Distribution

**Figure 2:**
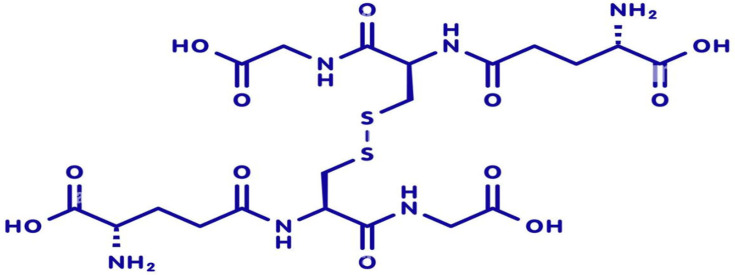
Molecular structure of oxidized glutathione (GSSG), with the chemical formula C_20_H_32_N_6_O_12_S_2_.

Glutathione is endogenously synthesized through a two-step ATP-dependent enzymatic process involving γ-glutamylcysteine synthetase (GCL) and glutathione synthetase (GS). The rate-limiting step is catalyzed by GCL, which combines glutamate and cysteine to form γ-glutamylcysteine, and is then converted into reduced glutathione (GSH) by GS. Cellular glutathione exists predominantly in its reduced form (GSH), which serves as a key antioxidant and redox buffer. Upon neutralizing reactive oxygen species (ROS), GSH is oxidized to glutathione disulfide (GSSG) (Figure 2). The enzyme glutathione reductase (GR) catalyzes the recycling of GSSG back to GSH using NADPH as a cofactor, maintaining the intracellular GSH/GSSG ratio—a critical indicator of oxidative stress and cellular health [15,16,17]. Glutathione is distributed across various cellular compartments, with high concentrations in the cytosol, mitochondria, and nucleus. Mitochondrial glutathione is crucial for regulating apoptosis and protecting energy-producing pathways from oxidative damage. In skin cells, glutathione supports keratinocyte proliferation, melanocyte regulation, and fibroblast-mediated extracellular matrix remodeling. Its compartmentalized distribution enables localized redox control, contributing to tissue-specific responses during aging and regeneration [18,19,20].

### 1.4. Role in Antioxidant Defense, Detoxification, and Redox Signaling

Glutathione plays a central role in maintaining cellular integrity through its multifaceted functions in antioxidant defense, detoxification, and redox signaling. As the most abundant intracellular thiol, reduced glutathione (GSH) directly scavenges reactive oxygen species (ROS) and reactive nitrogen species (RNS), thereby protecting cellular components from oxidative damage. Glutathione (GSH) acts as a key redox-sensitive regulator of chromatin structure and gene expression. Its influence on histones occurs through two complementary mechanisms: indirect metabolic control of histone methylation and direct, redox-dependent modification. Glutathione links cellular redox status to the epigenetic landscape by regulating the cell’s methylation potential. Histone S-glutathionylation is a direct epigenetic mechanism by which oxidative stress signals are transmitted to chromatin [21,22,23,24].

**Figure 3:**
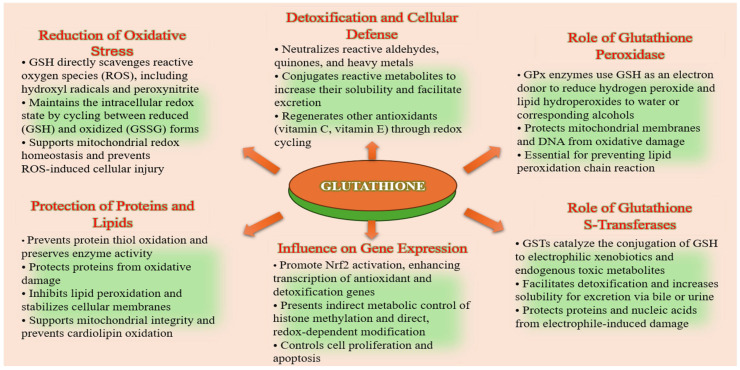
Role of glutathione in antioxidant defense, detoxification, and redox signaling.

It also serves as a cofactor for key antioxidant enzymes, such as glutathione peroxidase (GPx) and glutathione S-transferase (GST), that regulate redox homeostasis and detoxification. Glutathione S-transferases (GSTs) are an essential enzyme family that catalyzes the conjugation of GSH to electrophilic xenobiotics and endogenous toxic metabolites, thereby enhancing their solubility and promoting excretion [25,26,27,28,29]. The glutathione peroxidase (GPx) family of selenoenzymes catalyzes the reduction of hydrogen peroxide and lipid peroxides by using reduced glutathione (GSH) as an electron donor, thereby preventing oxidative damage to cellular membranes and proteins. During this process, GSH is oxidized to glutathione disulfide (GSSG), which is subsequently recycled back to its reduced form by glutathione reductase (GR) in an NADPH-dependent reaction. This enzymatic cycle maintains the intracellular GSH/GSSG ratio, which is a critical determinant of cellular redox status [30,31,32]. These enzymes are highly expressed in skin tissues and play a vital role in protecting keratinocytes, fibroblasts, and melanocytes from environmental insults, including ultraviolet (UV) radiation and pollutants [33,34]. The coordinated activity of GPx, GR, and GSTs underscores the central role of glutathione in antioxidant defense, cellular detoxification, and adaptive stress responses, making it a key molecular target in skin aging and regenerative therapies (Figure 3).

### 1.5. Glutathione in Skin Homeostasis

Glutathione plays a pivotal role in maintaining skin homeostasis and facilitating tissue regeneration through its antioxidant, anti-inflammatory, and cytoprotective properties. In the epidermis, glutathione regulates keratinocyte proliferation and differentiation, contributing to barrier integrity and cellular turnover. In the dermis, glutathione supports fibroblast viability and function, promoting collagen synthesis and extracellular matrix (ECM) remodeling, which are essential for structural resilience. Its presence helps preserve mitochondrial integrity and prevents apoptosis in regenerating cells [35,36,37]. Moreover, glutathione influences redox-sensitive signaling pathways, including MAPK, Nrf2, and TGF-β, which orchestrate cellular responses to damage and repair. In keratinocytes, glutathione supports proliferation and differentiation by maintaining redox balance and protecting against oxidative DNA damage. In fibroblasts, glutathione preserves mitochondrial integrity and prevents premature senescence, thereby sustaining their ability to synthesize extracellular matrix components, such as collagen and elastin [38,39]. Adequate intracellular levels of reduced glutathione (GSH) promote cell cycle progression, enhance epidermal regenerative capacity, and modulate inflammatory responses that influence barrier function. Under oxidative stress conditions, glutathione depletion impairs both keratinocyte turnover and fibroblast activity, contributing to delayed re-epithelialization, poor dermal remodeling, and visible signs of skin aging. During GSH depletion, blood cells are subjected to chronic oxidative stress [40,41,42]. Therapeutic strategies aimed at restoring glutathione levels have shown promise in enhancing cellular viability, promoting tissue regeneration, and improving clinical outcomes in dermatological applications.

### 1.6. Glutathione Influence on Melanogenesis and Pigmentation Balance

Glutathione plays a complex role in regulating melanogenesis and maintaining a balance in skin pigmentation. As a potent intracellular antioxidant, glutathione modulates the redox environment of melanocytes, which directly influences the activity of tyrosinase, the rate-limiting enzyme in melanin synthesis. Elevated levels of reduced glutathione (GSH) have been shown to inhibit tyrosinase activity and shift melanin production from the darker eumelanin pathway toward the lighter pheomelanin pathway, resulting in a skin-lightening effect [43,44,45]. Additionally, glutathione indirectly affects pigmentation by reducing oxidative stress, thereby stimulating melanogenesis via pro-inflammatory and redox-sensitive signaling pathways. Topical and systemic administration of glutathione has been associated with improved uniformity of skin tone and reduced hyperpigmentation, particularly in conditions such as melasma and post-inflammatory pigmentation. Overall, GSH’s regulatory effects on melanogenesis underscore its therapeutic potential in esthetic dermatology and pigmentary disorders [46,47,48].

### 1.7. Modulation of Wound Healing and Scar Formation

Glutathione is essential in coordinating the intricate cellular and molecular processes that drive wound healing and influence scar formation. During the initial inflammatory phase, glutathione mitigates oxidative stress by neutralizing reactive oxygen species (ROS), thereby limiting tissue damage and modulating the release of proinflammatory cytokines such as TNF-α and IL-1β. In the proliferative phase, it supports keratinocyte migration and re-epithelialization while preserving fibroblast viability and promoting extracellular matrix (ECM) synthesis [49,50,51]. Glutathione also enhances angiogenesis by protecting endothelial cells from oxidative injury and facilitating nitric oxide signaling [52,53].

During remodeling, it contributes to balanced collagen deposition and matrix turnover, thereby reducing the risk of hypertrophic scarring and fibrosis. By influencing redox-sensitive transcription factors, such as Nrf2 and TGF-β, glutathione helps regulate the expression of matrix metalloproteinases (MMPs) and tissue inhibitors (TIMPs), which are essential for controlled ECM remodeling. Glutathione deficiency or dysregulation has been associated with delayed wound closure, excessive inflammation, and aberrant scar formation [54,55].

Patient education represents an essential yet often overlooked component of optimal wound healing and scar management. Empowering individuals to understand the biological determinants of tissue repair—including the role of redox homeostasis and glutathione-dependent pathways—can significantly improve adherence to preventive strategies and post-injury care. Awareness of glutathione’s role in mitigating oxidative stress, modulating inflammation, and supporting extracellular matrix remodeling enables patients and clinicians to make informed decisions about topical agents, systemic supplementation, and lifestyle behaviors that influence the quality of healing. Integrating structured educational interventions into wound-care protocols may therefore enhance therapeutic outcomes, reduce maladaptive scarring, and promote long-term skin health by aligning clinical recommendations with a clear understanding of glutathione’s mechanistic relevance [56,57,58,59].

**Figure 4:**
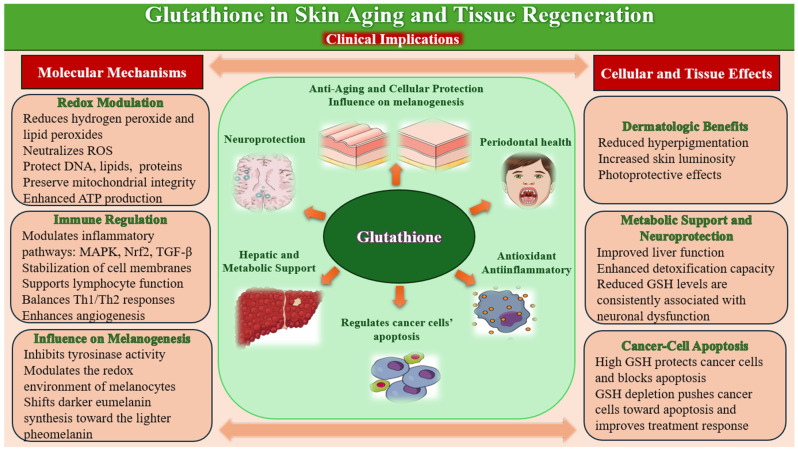
Glutathione: Molecular Mechanisms, Tissue Effects, and Clinical Implications.

Given its central role in redox regulation, detoxification, and cellular repair, glutathione has garnered increasing interest as a therapeutic agent in dermatology and regenerative medicine. Its applications span from skin brightening and anti-aging treatments to adjunctive support in wound healing and postprocedural recovery (Figure 4).

However, the clinical efficacy of glutathione depends on its delivery method, bioavailability, and stability.

### 1.8. Dietary Sources of Glutathione and Strategies to Enhance Its Bioavailability

Although glutathione (GSH) is synthesized endogenously in virtually all cells, dietary factors play a significant role in supporting its intracellular concentration and redox functionality. Glutathione is present in a variety of foods, but its direct bioavailability is limited by rapid hydrolysis by intestinal γ-glutamyltransferase. Consequently, nutritional strategies that provide precursors or enhance enzymatic synthesis are considered more effective than direct intake. Several foods contain reduced glutathione, particularly fresh fruits and vegetables. Among these, asparagus, avocado, spinach, zucchini, cucumber, green beans, and Brussels sprouts exhibit the highest concentrations. Although a substantial proportion of dietary GSH is degraded during digestion, these foods indirectly contribute to glutathione homeostasis through their antioxidant micronutrients and phytochemicals [60,61].

Endogenous glutathione synthesis depends on the availability of its constituent amino acids—cysteine, glutamate, and glycine. Cysteine is the rate-limiting substrate, making sulfur-containing foods particularly relevant. Eggs, poultry, fish, and lean meats provide bioavailable cysteine, while dairy products, legumes, nuts, and seeds contribute glycine and glutamate. Adequate intake of these amino acids enhances γ-glutamylcysteine synthetase activity and supports sustained GSH production. Cruciferous vegetables (e.g., broccoli, kale, cabbage, cauliflower) and allium species (garlic, onions, leeks) contain organosulfur compounds such as sulforaphane and allicin, which activate the Nrf2–ARE pathway. This transcriptional activation increases the expression of glutathione-synthesizing enzymes and promotes intracellular antioxidant defense. Broccoli sprouts, in particular, are recognized for their high sulforaphane content and potent stimulatory effect on phase II detoxification pathways [62,63,64].

Selenium is an essential cofactor for glutathione peroxidase, thereby supporting the enzymatic utilization and regeneration of GSH. Dietary sources include Brazil nuts, seafood (tuna, sardines, salmon), eggs, and whole grains. Additionally, vitamins C and E help maintain glutathione in its reduced form by participating in redox cycling and limiting oxidative damage. Fruits rich in vitamin C (citrus, berries, kiwi) and vitamin E–containing foods (almonds, sunflower seeds, cold-pressed oils) indirectly sustain intracellular GSH levels. Evidence indicates that specific dietary combinations—such as sulfur-rich vegetables with vitamin C, protein sources with polyphenol-rich plant foods, or sulforaphane-containing vegetables with myrosinase donors—synergistically enhance glutathione synthesis and bioefficacy [65,66].

Collectively, these nutritional strategies represent a feasible approach to optimizing glutathione status and improving antioxidant capacity.

## 2. Materials and Methods

### 2.1. Eligibility Criteria

#### 2.1.1. Inclusion Criteria

Studies were selected for inclusion if they met the following criteria:

(1) Articles written in English and available in full-text format were included.
(2) Studies that provided mechanistic insights, clinical outcomes, and therapeutic strategies in skin rejuvenation and regenerative therapies.
(3) Experimental (in vitro and in vivo) and clinical studies that investigated glutathione (GSH), oxidized glutathione (GSSG), the GSH/GSSG ratio, or associated redox pathways (e.g., Nrf2/Keap1, GCL, GSR, GGT) in the context of skin aging, photoaging, oxidative stress, extracellular matrix remodeling, mitochondrial function, cellular senescence, wound healing, or tissue regeneration.

#### 2.1.2. Exclusion Criteria

(1) Non-English articles;
(2) Research published before 2000;
(3) Studies unrelated to skin rejuvenation and regenerative therapies;
(4) Abstract-only publications or inaccessible sources;
(5) Studies reporting only global oxidative stress markers without a specific assessment of glutathione or redox-regulated pathways;
(6) Studies using cancer cell lines or tumor models were excluded unless they provided mechanistic insights into glutathione-mediated redox regulation applicable to normal skin physiology or regenerative processes.

### 2.2. Information Sources and Search Strategy

A comprehensive literature search was performed across six electronic databases: PubMed/MEDLINE, Scopus, Web of Science Core Collection, Embase, Cochrane Library, and Google Scholar. The search covered studies published between January 2000 and September 2025. The search strategy combined Medical Subject Headings (MeSH) and free-text terms using Boolean operators (AND, OR). The core search string applied in PubMed was: “glutathione”, “glutathione metabolism”, “redox homeostasis”, “skin rejuvenation”, “skin aging”, “tissue regeneration”, “wound healing”, “molecular mechanisms”, “cellular detoxification”, “bioavailability”, “topical delivery”, “clinical implications”, “ethical standards” and “pharmacovigilance”. Equivalent search strings were adapted for Scopus, Web of Science, Embase, and Cochrane Library according to each database’s indexing system.

### 2.3. Selection Process

Two independent reviewers screened the titles and abstracts of all identified studies to exclude irrelevant publications using predefined eligibility criteria. Full-text articles were obtained for all studies that passed the initial screening. The same two reviewers independently assessed the full texts to determine final eligibility. Any disagreements regarding study inclusion were resolved through discussion until consensus was reached. When necessary, a third reviewer was consulted to adjudicate unresolved discrepancies.

### 2.4. Data Collection Process and Data Items

To avoid merging heterogeneous study designs, the evidence was synthesized in separate analytical layers, with clinical, animal, and mechanistic in vitro studies analyzed independently. Clinical studies were evaluated as the primary source of human relevance, with a focus on glutathione-related outcomes in skin aging, photoaging, and wound healing. Animal studies were synthesized separately to elucidate tissue-level mechanisms, including extracellular matrix remodeling, mitochondrial dysfunction, inflammation, and regenerative capacity. Mechanistic in vitro studies were analyzed independently to characterize molecular pathways involving glutathione, redox homeostasis, senescence, and cellular responses relevant to cutaneous aging and tissue repair.

Data extraction was performed independently by two reviewers using a standardized data extraction form developed prior to the review process. All studies that met the eligibility criteria after full-text screening were included in the final synthesis (*n* = 194). Any discrepancies in extracted data were resolved through discussion until consensus was reached. For each included study, the following information was systematically collected:

(1) Study characteristics: author(s), year of publication, country, study design, sample size, and population type (human, animal, or in vitro);
(2) Intervention details: glutathione formulation, dosage, duration, and mode of administration (oral, topical, injectable, or experimental exposure);
(3) Outcomes measured: markers of skin aging and regeneration, including elasticity, hydration, collagen synthesis, oxidative stress parameters, photoprotection, and cellular repair indicators.

No authors were contacted for additional information, as all included studies provided sufficient methodological and outcome details for analysis.

### 2.5. Study Risk of Bias Assessment

The risk of bias of the included studies was assessed independently by two reviewers using validated tools appropriate for each study design. Randomized controlled trials were considered the highest level of evidence for clinical outcomes and were evaluated for randomization, blinding, allocation concealment, and completeness of reporting. Their methodological quality was assessed using the Cochrane Risk of Bias 2 (RoB 2) tool, which assesses five domains: (1) bias arising from the randomization process, (2) bias due to deviations from intended interventions, (3) bias due to missing outcome data, (4) bias in outcome measurement, and (5) bias in the selection of reported results. Non-randomized clinical studies, animal studies, and in vitro studies were assessed for methodological quality and risk of bias using standardized evaluation criteria. Each domain was rated as “Low”, “Some Concerns”, or “High” risk of bias. An overall high risk of bias was assigned when at least one domain was rated “High” or when multiple domains were rated “Some Concerns”. Disagreements between reviewers were resolved through discussion until consensus was reached.

### 2.6. Effect Measures and Data Synthesis

Given the heterogeneity of study designs, populations, interventions, and outcome measures, a narrative synthesis approach was employed. Effect measures extracted from the included studies consisted of reported changes in biochemical markers (e.g., oxidative stress parameters, collagen content, hydration levels), clinical outcomes (e.g., wrinkle depth, elasticity, pigmentation), and mechanistic indicators related to redox homeostasis and cellular detoxification. Studies were grouped thematically by mechanistic domains, including antioxidant activity, redox regulation, extracellular matrix remodeling, immune modulation, clinical implications, and delivery strategies (oral, topical, or experimental formulations). Findings from randomized controlled trials and interventional studies were summarized separately to highlight translational relevance. The synthesis emphasized identifying consistent trends, mechanistic pathways, and points of agreement or disagreement among the included studies.

### 2.7. Study Selection

The study selection process adhered to PRISMA 2020 guidelines. The database search yielded 2538 records: PubMed (*n* = 1393), Embase/Scopus (*n* = 348), Web of Science (*n* = 315), Cochrane Library (*n* = 273), and Google Scholar (*n* = 216). After removing 214 duplicates, 2324 records remained for screening. Of these, 669 were automatically marked as ineligible based on database filters, and 43 were excluded for other reasons (e.g., non-retrievable records, incomplete metadata). A total of 1619 records proceeded to title and abstract screening.

**Figure 5:**
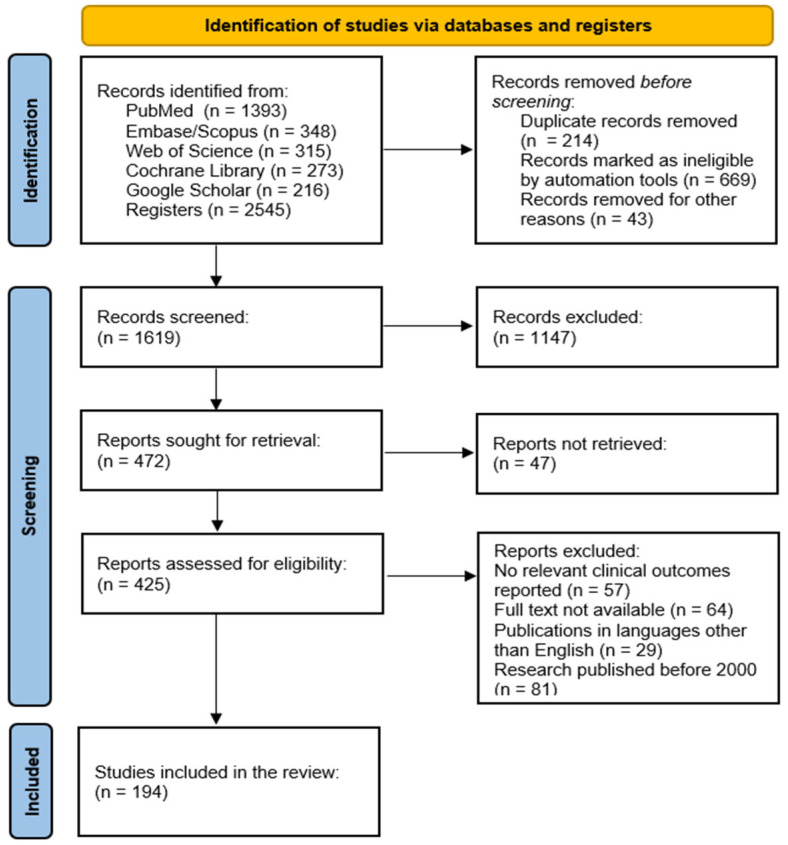
PRISMA flow diagram for identification of studies retrieved from databases.

During the screening phase, 1147 records were excluded for failing to meet the predefined eligibility criteria. The remaining 472 full-text articles were assessed for eligibility. Following a detailed evaluation, 194 studies met all inclusion criteria and were included in the final synthesis. Reasons for full-text exclusion were documented and are summarized in the PRISMA flow diagram (Figure 5).

Additional details are provided in the Supplementary Materials.

## 3. Results

A total of 194 studies met the inclusion criteria and were included in the final synthesis. The included studies comprised randomized controlled trials, non-randomized interventional studies, observational studies, in vivo experiments, and in vitro investigations. Study characteristics varied widely across design, population, intervention type, and outcome measures.

### 3.1. Glutathione and Tissue Regeneration

Although the skin represents a highly dynamic site of regeneration, its capacity to repair and remodel is tightly linked to systemic redox networks. Glutathione serves as a unifying molecular axis connecting cutaneous healing with cardiovascular, respiratory, digestive, and immune functions. This broader perspective highlights that tissue regeneration is part of a coordinated, organism-wide response.

#### 3.1.1. Glutathione and Cardiovascular Homeostasis

Glutathione plays a central role in maintaining cardiovascular integrity through its antioxidant, anti-inflammatory, and metabolic regulatory functions. Endothelial cells rely heavily on intracellular GSH to preserve nitric oxide (NO) bioavailability, prevent eNOS uncoupling, and limit peroxynitrite formation. With advancing age, endothelial cells exhibit reduced nitric oxide (NO) bioavailability, increased oxidative stress, and impaired barrier function [67,68,69]. These changes promote vasoconstriction, leukocyte adhesion, and low-grade inflammation, collectively diminishing microvascular responsiveness. In the vascular wall, GSH prevents the oxidative modification of low-density lipoproteins (LDL), a key initiating event in atherogenesis [70,71,72]. By modulating redox-sensitive transcription factors such as NF-κB and Nrf2, GSH influences vascular inflammation, smooth muscle proliferation, and plaque stability [73,74]. In ischemia–reperfusion injury, GSH depletion exacerbates mitochondrial dysfunction, ROS bursts, and cardiomyocyte apoptosis, whereas GSH restoration improves post-ischemic recovery and reduces infarct size in experimental models. Pericyte loss and altered smooth muscle tone further compromise vessel stability and autoregulation. Consequently, tissues experience reduced oxygen delivery, impaired nutrient exchange, and reduced removal of metabolic by-products. Across in vitro and in vivo models, GSH depletion consistently increases ROS accumulation, mitochondrial dysfunction, and oxidative DNA/protein damage, while GSH supplementation restores redox balance and prevents UV-induced oxidative stress. S-glutathionylation has emerged as a reversible regulatory mechanism that modulates protein activity under oxidative stress. Adequate GSH levels support vasodilation, microvascular perfusion, and barrier stability, thereby counteracting early endothelial dysfunction [75,76,77,78,79,80].

Collectively, these mechanisms position glutathione as a crucial determinant of cardiovascular resilience, with implications for hypertension, atherosclerosis, heart failure, and microcirculatory aging.

#### 3.1.2. Glutathione in Respiratory Defense and Pulmonary Redox Balance

The lungs are a major interface between the organism and the external environment, continuously exposed to oxygen, pollutants, and airborne oxidants. Because of this unique exposure, pulmonary redox balance plays a central role in shaping systemic oxidative status. Adequate GSH levels in the epithelial lining fluid maintain barrier integrity, regulate immune activation, and prevent oxidative injury to alveolar macrophages. When the antioxidant defenses of the respiratory epithelium, particularly glutathione, are intact, the lungs act as a metabolic buffer, limiting the spillover of reactive oxygen species (ROS) into the circulation [81,82,83,84]. This systemic protection preserves endothelial function, maintains microvascular perfusion, and supports the metabolic conditions required for efficient tissue regeneration. Excess ROS generated in the lungs can diffuse into the bloodstream or trigger inflammatory signaling cascades that propagate to distant tissues. Alveolar macrophages depend on GSH to sustain phagocytic activity, cytokine balance, and pathogen clearance. These circulating mediators impair mitochondrial function, reduce nitric oxide bioavailability, and disrupt redox-sensitive pathways essential for cell proliferation, migration, and extracellular matrix remodeling. Most studies converge on GSH’s role in suppressing NF-κB activation, reducing pro-inflammatory cytokine production (IL-1β, IL-6, TNF-α), and enhancing Nrf2-dependent antioxidant gene expression [85,86].

When pulmonary GSH is depleted, whether due to aging, pollution, smoking, or chronic respiratory disease, the resulting redox imbalance contributes to systemic inflammation and endothelial dysfunction, both of which hinder regenerative processes in the skin and other organs. As a result, tissues exhibit delayed healing, reduced angiogenic capacity, and increased susceptibility to fibrosis. Conversely, GSH augmentation, either through precursors or redox-active compounds, has been shown to improve mucociliary function, reduce oxidative burden, and modulate airway hyperresponsiveness [87,88,89,90].

These findings underscore the systemic relevance of glutathione in maintaining respiratory homeostasis and mitigating oxidative lung injury. By modulating oxidative load, inflammatory tone, and microvascular health, the respiratory system indirectly determines the efficiency and quality of regeneration across the body.

#### 3.1.3. Glutathione and Gastrointestinal Integrity

Gastrointestinal integrity and the composition of the gut microbiome exert profound systemic effects that directly shape tissue regeneration. The intestinal epithelium is the most rapidly renewing tissue in the human body, maintained by a tightly regulated interplay between epithelial cells, mesenchymal stromal cells, immune mediators, and the resident microbiota. This dynamic environment not only preserves barrier function but also generates metabolic and immunological signals that influence regenerative processes throughout the organism [91,92].

A healthy intestinal barrier prevents the translocation of microbial products and dietary antigens, maintaining immune tolerance and limiting systemic inflammation. Mesenchymal stromal cells—including fibroblasts, myofibroblasts, and pericytes—further support epithelial renewal by modulating intestinal stem cell activity through WNT, BMP, and R-spondin signaling pathways. In wound-healing models, GSH enhances re-epithelialization, modulates fibroblast–myofibroblast transition, and supports balanced ECM deposition. When barrier integrity is compromised, these regulatory networks become dysregulated, leading to chronic inflammation and impaired regenerative capacity. The microbiome also influences tissue regeneration through metabolic outputs such as short-chain fatty acids (SCFAs), which enhance epithelial repair, modulate immune responses, and support mitochondrial function in distant tissues. Conversely, dysbiosis increases oxidative stress, disrupts epithelial homeostasis, and promotes systemic inflammatory signaling that interferes with angiogenesis, fibroblast activity, and extracellular matrix remodeling [93,94].

Within the gastrointestinal tract, glutathione is essential for maintaining epithelial barrier function, regulating mucosal immunity, and supporting detoxification processes. Enterocytes exhibit high GSH turnover due to constant exposure to dietary oxidants, xenobiotics, and microbial metabolites. Adequate GSH levels preserve tight junction integrity, limit lipid peroxidation, and prevent translocation of luminal antigens. GSH also shapes host–microbiome interactions by modulating redox-sensitive signaling pathways and influencing microbial composition. In inflammatory bowel diseases, GSH depletion correlates with increased oxidative stress, impaired mucosal healing, and dysregulated immune responses. Furthermore, the gastrointestinal tract is a major site of absorption for glutathione precursors—particularly cysteine and glycine—linking digestive health directly to systemic redox capacity [95,96,97].

Through these mechanisms, glutathione contributes to digestive resilience, nutrient assimilation, and systemic metabolic homeostasis.

#### 3.1.4. Glutathione and Neurodegeneration

Neurodegenerative diseases such as Alzheimer’s, Parkinson’s, and ALS are strongly associated with accelerated cellular senescence. Senescent cells accumulate in neural and non-neural tissues and secrete a pro-inflammatory SASP (senescence-associated secretory phenotype) that disrupts stem cell niches, inhibits proliferation, and reduces the ability of tissues to repair damage. Neurodegeneration triggers persistent activation of microglia and astrocytes, leading to the release of cytokines and ROS that enter the systemic circulation. This inflammatory spillover impairs angiogenesis, fibroblast function, and extracellular matrix remodeling—key steps in tissue regeneration. Common mechanisms in neurodegenerative diseases—oxidative stress, mitochondrial dysfunction, and impaired protein homeostasis—deplete metabolic resources available for regeneration [98,99].

Glutathione (GSH) is the brain’s most abundant intracellular antioxidant and a central regulator of neuronal redox homeostasis. Neurons are particularly vulnerable to oxidative stress due to their high metabolic rate, intense mitochondrial activity, and limited capacity to upregulate antioxidant defenses. When GSH levels decline, the nervous system becomes increasingly susceptible to oxidative injury, mitochondrial dysfunction, and protein misfolding—hallmarks of neurodegenerative diseases. Reduced GSH availability has been consistently linked to neuronal loss in aging and in major neurodegenerative disorders, including Parkinson’s disease, Alzheimer’s disease, Huntington’s disease, amyotrophic lateral sclerosis, multiple sclerosis, and stroke. In these conditions, impaired GSH metabolism disrupts the detoxification of reactive oxygen and nitrogen species, leading to lipid peroxidation, DNA damage, and activation of apoptotic pathways. GSH depletion also promotes the aggregation of misfolded proteins, a key pathogenic mechanism in neurodegenerative disease, and accelerates mitochondrial ROS production, loss of membrane potential, and activation of senescence markers (p16, p21, SA-β-gal) [100,101,102,103].

**Figure 6:**
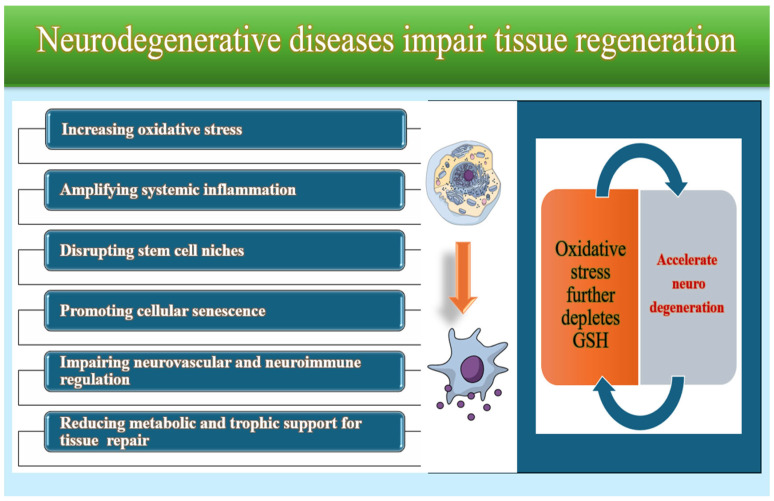
Common mechanisms in neurodegenerative diseases.

Mitochondria are especially affected by GSH imbalance. Loss of mitochondrial GSH impairs electron transport chain function, increases ROS production, and triggers neuronal energy failure. This creates a self-amplifying cycle in which oxidative stress further depletes GSH, accelerating neurodegeneration (Figure 6). Clinical studies show that patients with neurodegenerative diseases have significantly lower GSH levels in plasma and red blood cells than healthy individuals [104,105,106].

Overall, glutathione acts as a critical neuroprotective molecule. Its depletion is not merely a consequence of neurodegeneration but an early and potentially causative event that drives oxidative stress, inflammation, and neuronal death. Strategies aimed at restoring GSH levels or enhancing its metabolic pathways are therefore being explored as therapeutic approaches to slow or prevent the progression of neurodegenerative disease.

Systemic findings were crucial for elucidating mechanisms that have downstream effects on cutaneous physiology and tissue regeneration. The synthesis indicates that glutathione-dependent redox pathways across major organ systems modulate skin homeostasis, inflammatory signaling, extracellular matrix dynamics, and wound-healing capacity.

#### 3.1.5. Glutathione as a Protective Factor in Carcinogenesis

Glutathione (GSH) plays a fundamental protective role in the early stages of carcinogenesis by maintaining genomic stability, detoxifying electrophilic compounds, and buffering oxidative stress. As the most abundant intracellular thiol, GSH neutralizes reactive oxygen and nitrogen species that would otherwise induce DNA strand breaks, base modifications, and chromosomal instability, key initiating events in malignant transformation. Cutaneous carcinogenesis is a multistep process driven by the interaction between environmental carcinogens, particularly ultraviolet (UV) radiation, and the intrinsic defense mechanisms of skin cells. Glutathione (GSH), the most abundant intracellular antioxidant, plays a central role in protecting keratinocytes and melanocytes from malignant transformation. Through the activity of glutathione peroxidases and glutathione S-transferases, GSH participates in the detoxification of lipid peroxides, xenobiotics, and environmental carcinogens, thereby reducing mutational burden and preserving cellular integrity [107,108,109].

Beyond its antioxidant capacity, GSH modulates redox-sensitive signaling pathways that govern cell proliferation, apoptosis, and immune surveillance. Adequate GSH levels support the proper function of p53, maintain mitochondrial membrane stability, and prevent the activation of pro-oncogenic transcription factors such as NF-κB under basal conditions. By sustaining a balanced intracellular redox environment, GSH helps ensure that damaged cells undergo controlled apoptosis rather than progressing toward malignant transformation [110,111].

GSH also helps maintain tissue microenvironments that resist tumor initiation. In epithelial and stromal compartments, sufficient GSH availability limits chronic inflammation, reduces cytokine-driven oxidative injury, and preserves the integrity of extracellular matrix components. These effects collectively create a biochemical landscape that is less permissive to DNA damage, aberrant cell signaling, and early neoplastic changes [112,113,114]. Thus, glutathione functions as a central guardian against carcinogenesis, acting at multiple molecular and cellular levels to prevent the transition from normal tissue homeostasis to malignant initiation. Its protective role underscores the importance of systemic redox balance in maintaining genomic stability and reducing cancer risk.

### 3.2. Implications for Dermatology, Pharmacology, and Skin Care Innovations

Glutathione’s expanding clinical relevance carries significant implications for dermatology, pharmacology, and the future of skin care innovation. In dermatology, its function as a redox modulator and pigmentation regulator makes it a versatile agent for the treatment of pigmentary disorders, photoaging, and inflammatory dermatoses. Glutathione has become a fundamental component of integrative anti-aging protocols and post-procedural recovery strategies due to its powerful antioxidant, anti-inflammatory, and detoxifying properties. From a pharmacological perspective, glutathione exemplifies the challenges and opportunities of delivering bioactive molecules, prompting advances in formulation science, nanotechnology, and transdermal systems to overcome stability and bioavailability barriers. Advances in clinical pharmacology are increasingly integrating artificial intelligence-driven approaches to optimize drug discovery, personalized medicine, and pharmacovigilance, in which glutathione pathways could be crucial targets [115,116,117,118]. These innovations are driving the development of liposomal carriers, microneedle patches, and redox-sensitive hydrogels tailored for dermatological applications. In the skin care industry, glutathione has catalyzed the development of a new generation of multifunctional products that combine antioxidant defense with pigmentation control, hydration, and barrier support [119,120]. As research continues to refine its mechanisms and optimize its delivery, glutathione is positioned to become a fundamental component of integrative dermatological care and a catalyst for innovation in both clinical and cosmetic fields.

Glutathione has been formulated in various delivery systems to address skin aging, pigmentation disorders, and oxidative stress-related dermatoses. Among these, the topical, oral, and injectable routes are the most widely explored, each with distinct pharmacokinetic profiles, clinical applications, and limitations.

### 3.3. Clinical Studies Supporting Glutathione’s Dermatological Use

Clinical studies evaluating oral, topical, or injectable glutathione formulations reported heterogeneous outcomes, though generally positive. Commonly assessed parameters included skin brightness, pigmentation, hydration, elasticity, wrinkle depth, and post-procedural recovery. Variability in formulation, dosage, delivery modalities, and treatment duration contributed to differences in reported outcomes (Table 1).

#### 3.3.1. Topical Glutathione

Topical glutathione formulations are designed to deliver antioxidants directly to the skin, targeting localized oxidative damage and pigmentation irregularities. These products often incorporate liposomal, nanoemulsion, or gel-based carriers to enhance dermal penetration and protect glutathione from degradation by the skin. Topical application has shown promise in improving skin brightness, reducing melanin synthesis, and attenuating the signs of photoaging. However, the stratum corneum presents a significant barrier to effective transdermal delivery, and maintaining the stability of glutathione in aqueous formulations remains challenging. Recent innovations, such as dual-phase systems and encapsulated glutathione derivatives, have demonstrated improved bioavailability and clinical efficacy in small-scale studies [121,122,123].

Multiple randomized controlled trials and systematic reviews have investigated the dermatological benefits of glutathione, particularly its effects on skin tone, elasticity, and texture. Topical application of glutathione has been shown to significantly enhance skin brightness, texture, and resilience. In a double-blind study, Watanabe et al. (2014) demonstrated that oxidized glutathione significantly improved skin tone and reduced hyperpigmentation over 10 weeks. The study found that skin brightness and moisture content improved significantly in the treatment group compared with the placebo group, and hyperpigmentation was visibly reduced. No serious adverse effects were reported, indicating good tolerability and safety. The formulation was stable and effective in delivering glutathione to the epidermis [124].

Grandi et al. (2019) conducted a randomized trial that revealed a significant reduction in UVB-induced erythema and an improvement in skin texture with the use of 2% S-acyl glutathione cream. In this randomized trial, the effects of 2% S-acyl glutathione cream on UVB-irradiated skin were evaluated, with a focus on its anti-inflammatory and barrier-repair properties. The S-acylated form of glutathione used in this trial is notable for its enhanced stability and lipophilicity, allowing better skin penetration and intracellular delivery than reduced glutathione. These results support its use in post-sun exposure care, anti-aging regimens, and formulations for sensitive or inflamed skin [125].

Cui et al. (2024) found that topical glutathione precursors shielded the skin from oxidative and environmental stress, indicating their potential to enhance the skin barrier. In this preclinical-to-clinical translational study, Cui et al. investigated the impact of topical glutathione precursors, notably N-acetylcysteine (NAC) and glycine, on skin exposed to oxidative and environmental stressors, including UV radiation and urban pollutants. This study supports the concept that precursor-based topical strategies can stimulate endogenous glutathione production in situ, offering a noninvasive, stable, and bioavailable alternative to direct glutathione application. This reinforces glutathione’s role in barrier repair and environmental defense, making it a compelling candidate for urban skin care formulations and as an adjunctive therapy for inflammatory dermatoses [126].

These trials established topical glutathione as a viable option for cosmetic skin brightening and laid the groundwork for further studies exploring liposomal and nanoemulsion-based delivery systems.

#### 3.3.2. Oral Glutathione

Oral supplementation with glutathione is widely used for systemic antioxidant support and skin lightening. Available in reduced, S-acetylated, or liposomal forms, oral glutathione is intended to elevate plasma and tissue glutathione levels via gastrointestinal absorption. However, its effectiveness is limited by enzymatic degradation in the gut and by first-pass hepatic metabolism.

A double-blind, placebo-controlled study conducted by Arjinpathana and Asawanonda in 2010 demonstrated that oral glutathione at 500 mg/day significantly reduced the melanin index after 4 weeks, supporting its efficacy as a depigmenting agent. This study used objective spectrophotometric measurements to quantify pigmentation changes, enhancing methodological rigor. The results suggest that oral glutathione may inhibit melanogenesis by suppressing tyrosinase activity and modulating antioxidant activity, although bioavailability remains a limiting factor. No serious adverse effects were reported, confirming a favorable safety profile [127].

Handog et al. (2016) conducted an open-label trial involving Filipino women, demonstrating that oral glutathione administration produced noticeable skin-lightening effects. Individual responses varied, with some showing marked improvement and others showing minimal change, highlighting the variability in absorption, metabolism, and baseline oxidative status. This study emphasized patient satisfaction, with most participants reporting improved skin radiance and tone. No serious adverse events were recorded, reinforcing the favorable safety profile of oral glutathione [128].

Duperray et al. (2021) investigated the skin-lightening and anti-dark-spot effects of an oral combination of L-Cystine and reduced L-Glutathione (GSH) compared with placebo and benchmark treatments. It was designed as a 12-week, randomized, double-blind, parallel-group, benchmark- and placebo-controlled clinical trial involving 124 Asian women. The study demonstrates that oral supplementation with L-Cystine combined with reduced L-Glutathione is an effective and safe approach for lightening overall skin tone and reducing the size and intensity of facial dark spots [129].

The study by Richie et al. (2014) is among the most frequently cited clinical trials evaluating whether oral glutathione (GSH) supplementation increases glutathione levels in humans. It provides strong clinical evidence that oral glutathione supplementation is effective, dose-dependent, and safe for increasing glutathione levels in humans. It also demonstrates beneficial effects on reducing oxidative stress and enhancing immune function. The findings support the use of oral GSH as a viable strategy to boost systemic antioxidant capacity [130].

The article by Weschawalit et al. (2017) provides a comprehensive review of the biological roles of glutathione (GSH) and evaluates its clinical effects on skin pigmentation and aging. The authors discuss both mechanistic pathways and clinical evidence, highlighting the growing relevance of glutathione in cosmetic dermatology [131].

In summary, topical glutathione is ideal for addressing localized hyperpigmentation, melasma, or post-inflammatory dark spots, while oral glutathione is more suitable for overall skin brightening and antioxidant support, and for patients seeking effects on the entire face or body. This highlights the potential of oral glutathione supplementation in cosmetic dermatology.

#### 3.3.3. Injectable Glutathione

Injectable glutathione, typically administered intravenously, offers the highest bioavailability because it bypasses the gastrointestinal tract. Intravenous glutathione is often used in cycles, with dosing protocols that vary across clinical settings. While some studies suggest improvements in skin clarity, elasticity, and pigmentation, the evidence remains limited and inconsistent. Moreover, concerns regarding safety, potential adverse reactions, and lack of regulatory approval in certain regions have prompted calls for more rigorous clinical evaluations. Several regulatory bodies, including the FDA and the Philippine FDA, have issued warnings in response to reports of severe adverse events, such as renal failure, thyroid dysfunction, and Stevens–Johnson syndrome. Current evidence strongly discourages intravenous administration for cosmetic purposes [132,133,134,135].

The article by Sonthalia et al. (2016) addresses the controversies and regulatory warnings surrounding injectable glutathione, despite anecdotal evidence of rapid skin depigmentation. However, these effects are often unverified in controlled clinical trials, and outcomes vary significantly across individuals. Ethical concerns have been raised regarding the promotion of skin-whitening treatments in contexts where colorism and social pressure may influence patient decisions. This review calls for culturally sensitive guidelines and transparent patient education. The review highlights that although glutathione may inhibit tyrosinase and thereby reduce melanin synthesis, the systemic effects of high-dose intravenous administration on redox balance and organ function remain poorly understood [132].

Sarkar et al. (2024) conducted a comprehensive systematic review evaluating the efficacy, mechanisms, formulations, and safety of glutathione (GSH) for skin lightening and melasma. The review synthesizes evidence from oral, topical, sublingual, and intravenous formulations, highlighting both therapeutic potential and limitations [134].

Clinical outcomes associated with glutathione supplementation vary substantially according to the route of administration, reflecting differences in bioavailability, pharmacokinetics, tissue penetration, and metabolic stability. Across published clinical studies, three major delivery pathways—oral, topical, and parenteral—demonstrate distinct therapeutic profiles and magnitudes of effect (Table 2).

Ongoing research into advanced delivery systems, such as microneedle patches, transdermal hydrogels, and glutathione-loaded nanoparticles, holds promise for overcoming current limitations and optimizing clinical outcomes in dermatological applications.

#### 3.3.4. Advances in Delivery Systems: Liposomes, Nanoparticles, Hydrogels

Recent innovations in drug delivery systems have significantly enhanced the therapeutic potential of glutathione in dermatology by improving its stability, bioavailability, and skin penetration. Liposomes, spherical vesicles composed of phospholipid bilayers, have emerged as effective carriers for topical glutathione due to their biocompatibility and ability to encapsulate both hydrophilic and lipophilic molecules. Liposomal formulations protect glutathione from oxidative degradation and facilitate transdermal absorption, particularly when combined with penetration enhancers or emulsifiers. Nanoparticles, including polymeric and lipid-based nanocarriers, offer controlled release profiles and targeted delivery to specific skin layers. Their small size allows deeper penetration into the epidermis and dermis, while surface modifications can improve cellular uptake and reduce systemic clearance [136,137,138,139,140]. Hydrogels, three-dimensional polymeric networks that retain large amounts of water, provide a versatile platform for sustained release of glutathione (GSH) in wound-healing and regenerative applications. Thermoresponsive and pH-sensitive hydrogels can be engineered to respond to local skin conditions, thereby enhancing therapeutic precision [141,142,143]. These advanced delivery systems not only address the traditional limitations of glutathione administration but also create opportunities to develop personalized, minimally invasive dermatological therapies [144,145]. Future directions emphasize the need for longitudinal multicenter trials, biomarker-guided personalization, and expanded roles in regenerative medicine and dermatological oncology. Collectively, these findings position glutathione as a promising agent for integrative skin care and therapeutic innovation, warranting continued research and evidence-based refinement.

### 3.4. Differentiation Between Medical and Esthetic Indications

The clinical use of glutathione spans two distinct domains—medical indications, where treatment is grounded in pathophysiology and therapeutic necessity, and esthetic indications, where the primary goal is cosmetic enhancement. Differentiating between these contexts is essential for establishing appropriate dosing, safety monitoring, ethical standards, and regulatory compliance.

#### 3.4.1. Medical Indications

Medical applications of glutathione focus on restoring redox balance, mitigating oxidative stress, and supporting detoxification pathways. Evidence-based indications include:

− Hepatic disorders (e.g., nonalcoholic fatty liver disease, drug-induced hepatotoxicity), which involve lipid accumulation and oxidative stress in hepatocytes. Glutathione replenishment supports antioxidant defense and conjugation pathways [146].
− Neurodegenerative conditions such as Parkinson’s disease, in which glutathione depletion contributes to mitochondrial dysfunction and dopaminergic neuron vulnerability [98,99,103].
− In diabetes, glutathione depletion is linked to β-cell dysfunction and the pathogenesis of diabetic complications [147].
− Periodontal health is another area where glutathione’s antioxidant and anti-inflammatory properties are clinically relevant. In periodontitis, reduced glutathione levels correlate with increased oxidative damage and inflammation [55,56].
− Chronic inflammatory or metabolic disorders, where glutathione modulates cytokine activity and oxidative biomarkers [21,26].
− Glutathione also plays a crucial role in oncology. It functions as an antioxidant and detoxifying agent, protecting cells from carcinogens and oxidative damage [108,148,149].
− Glutathione influences the efficacy and toxicity of chemotherapy through its antioxidant and detoxifying properties. It is employed as an adjunctive therapy to mitigate chemotherapy-induced toxicity, particularly in reducing nephrotoxicity associated with cisplatin [150,151].
− Beyond direct therapeutic applications, glutathione’s unique biochemical properties make it valuable in biomedicine, particularly for developing functional metal nanomaterials for biosensing, bioimaging, and anticancer therapies such as photothermal and radiotherapy. Its role as a protective ligand and reducing agent enhances the biocompatibility and functional performance of these nanomaterials [152].

In these contexts, glutathione is used to correct documented biochemical deficits, and treatment is guided by clinical biomarkers, standardized dosing, and medical supervision.

#### 3.4.2. Esthetic Indications

Esthetic applications of glutathione, including skin lightening, brightening, and anti-aging, are driven by cosmetic rather than medical needs. Mechanisms include the inhibition of tyrosinase and melanogenesis, the modulation of pheomelanin/eumelanin ratios, and the reduction of oxidative stress in photoexposed skin [124,133]. While oral and topical formulations have demonstrated modest improvements in pigmentation and skin quality, the evidence for injectable glutathione in esthetic settings remains inconsistent [134].

Ongoing research continues to expand the clinical relevance of glutathione and optimize therapeutic strategies that use it.

#### 3.4.3. Ethical and Regulatory Considerations

The distinction between medical and esthetic indications carries important ethical implications. Cosmetic use, especially for skin lightening, may be influenced by sociocultural pressures, colorism, and unrealistic beauty standards. Clinicians are encouraged to provide transparent counseling, ensure informed consent, and avoid promoting unvalidated or potentially harmful interventions. Regulatory frameworks generally support medical indications with documented clinical benefit, while esthetic applications often fall outside approved therapeutic use [132,153,154].

Understanding the difference between medical and esthetic indications is critical for guiding clinical decision-making, ensuring patient safety, and maintaining ethical standards. While glutathione has validated roles in several medical conditions, its esthetic applications require cautious interpretation of evidence, strict adherence to regulatory guidelines, and culturally sensitive patient communication.

## 4. Discussion

The findings of this systematic review indicate that glutathione plays a multifaceted role in tissue regeneration, with consistent evidence supporting its involvement in modulating oxidative stress, preserving the extracellular matrix, and promoting cellular repair. Clinical and experimental studies converge on the conclusion that glutathione improves skin quality by reducing oxidative biomarkers, enhancing hydration and elasticity, and attenuating pigmentation irregularities. These effects align with established mechanistic pathways, including redox homeostasis, detoxification, and melanogenesis regulation. Although the magnitude of clinical benefit varies across studies, the overall pattern suggests that glutathione may support tissue regeneration by stabilizing the cellular microenvironment and promoting balanced inflammatory responses [35,49].

### 4.1. Synergistic Use with Other Antioxidants

Glutathione’s therapeutic efficacy in dermatology is significantly enhanced when used in combination with other antioxidants, particularly vitamin C and niacinamide. Vitamin C (ascorbic acid) acts as a potent free radical scavenger, stabilizing and regenerating glutathione, thereby amplifying its intracellular activity and prolonging its antioxidant effects. This synergy improves skin brightness, reduces oxidative stress, and supports collagen synthesis, making this combination especially effective for treating hyperpigmentation, photoaging, and post-inflammatory discoloration. Niacinamide (vitamin B3), another well-established antioxidant, complements glutathione by improving the epidermal barrier function, reducing transepidermal water loss, and modulating inflammatory cytokine production. It also inhibits melanosome transfer from melanocytes to keratinocytes, thereby enhancing the depigmenting effects of glutathione. Clinical protocols often pair glutathione with vitamin C, administered orally or topically for optimal results. These combinations not only improve antioxidant resilience but also offer broader benefits, including improved skin tone, uniformity, elasticity, and overall dermal health [155,156,157,158,159,160,161].

Combination therapy downregulated nicotinamide nucleotide transhydrogenase (NNT), a key mitochondrial enzyme involved in redox homeostasis, thereby reducing melanin synthesis and oxidative damage. Melanogenesis is the biological process by which melanin is synthesized in specialized cells called melanocytes in the skin. This process occurs inside melanosomes, where melanogenic enzymes such as tyrosinase and tyrosinase-related proteins catalyze melanin production. Melanogenesis was significantly inhibited, as evidenced by reduced tyrosinase and microphthalmia-associated transcription factor (MITF) expression in melanocyte cultures. In vivo application led to visible improvements in skin tone uniformity and radiance, with high tolerability and no adverse effects. Polydeoxyribonucleotide (PDRN) contributes significantly to tissue repair and exhibits notable anti-inflammatory effects. It promotes tissue regeneration primarily by activating the adenosine A2A receptor, which in turn stimulates cellular processes essential for wound healing and repair, including collagen synthesis and angiogenesis. This highlights the importance of redox pathway modulation as a therapeutic target for pigmentary and aging-related skin conditions [162,163,164,165,166,167].

Park et al. (2022) studied niacinamide, vitamin C, and PDRN, demonstrating synergistic antioxidant effects and a reduction in melanogenesis by modulating NNT and redox pathways. Synergistic antioxidant activity was observed, with enhanced scavenging of reactive oxygen species (ROS) and improved cellular redox balance [168].

Wahab et al.’s (2021) study is a pivotal contribution to the evidence supporting the use of combination glutathione therapy in esthetic dermatology. In this double-blind, randomized controlled trial, the researchers evaluated the effects of the oral glutathione alone, topical glutathione alone, and a combination of oral and topical glutathione. The group receiving the combination treatment showed significantly greater reductions in the melanin index and greater uniformity of skin tone than either monotherapy group. It also highlights the importance of formulation quality, dose consistency, and treatment duration in achieving optimal results [169].

Although the evidence remains heterogeneous, the synergistic potential of these combinations highlights the importance of integrated antioxidant strategies in dermatologic interventions. Further controlled trials are needed to clarify optimal dosing, timing, and formulation synergies.

### 4.2. Issues with Bioavailability, Stability, and Standardization

Despite its therapeutic potential, glutathione faces formulation and clinical challenges related to its bioavailability, stability, and standardization that limit its widespread use in dermatology. Bioavailability remains a significant concern, particularly with oral supplementation, as intestinal enzymes rapidly degrade glutathione, which undergoes extensive first-pass hepatic metabolism. Differences in bioavailability and safety profiles across delivery modalities are critical when evaluating the clinical relevance of glutathione. Intravenous administration provides markedly higher systemic bioavailability but is associated with short-lived effects and potential safety concerns, particularly in individuals with comorbidities. In contrast, oral glutathione is generally safe yet exhibits low absorption unless delivered in liposomal formulations that protect it from gastrointestinal degradation. Alternative oral strategies, such as N-acetylcysteine (NAC), offer improved stability and precursor-based replenishment of intracellular GSH, enabling more efficient transport across biological barriers. This limits systemic absorption and necessitates the use of modified forms, such as S-acetyl glutathione or liposomal encapsulation, to enhance its delivery. Stability is another major issue: glutathione is highly sensitive to oxidation, light, and temperature fluctuations, which can diminish its antioxidant capacity before it reaches target tissues. Innovative formulations such as proliposomes and niosomes have been developed to enhance the oral bioavailability and stability of glutathione by protecting it from degradation and prolonging its release, with promising results in preclinical studies [170,171,172,173].

Glutathione topical formulations often require protective carriers to improve their stability, bioavailability, and skin penetration. Because of glutathione’s susceptibility to degradation and limited skin permeability, encapsulation in carriers such as lipid-based nanoparticles, liposomes, or nanostructured lipid carriers (NLCs) enhances its stability and facilitates controlled release at the site of application. These carriers protect glutathione from oxidation and enzymatic degradation, improving its antioxidant efficacy in topical use [174,175].

Standardization across delivery routes—oral, topical, and injectable—is lacking, with wide variability in dosing protocols, treatment duration, and product quality. Addressing these challenges through advanced formulation technologies, biomarker-guided dosing, and rigorous clinical trials is essential to optimize glutathione’s therapeutic potential and ensure its safe, evidence-based integration into dermatological practice.

### 4.3. Controversies Surrounding Glutathione’s Expanding Clinical Relevance and Molecular Mechanism

At the molecular level, the compartmentalization of glutathione in cellular organelles such as mitochondria, the nucleus, and the endoplasmic reticulum adds complexity to understanding its precise regulatory roles and involvement in pathways beyond antioxidant defense, including cellular proliferation, apoptosis, and signal transduction. This multi-compartment presence challenges straightforward interpretations of its molecular actions and therapeutic targeting [17,19].

While glutathione is widely recognized for its antioxidant and detoxifying roles, its expanding clinical use, particularly in esthetic dermatology, has sparked ongoing debate. One major controversy lies in the incomplete understanding of the molecular mechanisms underlying this process in human skin. Although glutathione modulates melanogenesis, inflammation, and oxidative stress through pathways such as tyrosinase inhibition and Nrf2 activation, its precise intracellular dynamics, tissue-specific effects, and long-term consequences remain insufficiently characterized. This mechanistic ambiguity complicates the development of targeted therapies and raises concerns regarding off-label use. These concerns are especially pertinent in the case of unregulated injectable formulations, which have been associated with potential renal and thyroid dysfunction and the risk of masking underlying dermatologic or systemic conditions [20,132].

Clinically, the widespread promotion of glutathione for skin lightening, especially via intravenous administration, has drawn criticism from regulatory bodies and medical societies. Despite anecdotal reports and small-scale studies suggesting cosmetic benefits, robust evidence from large controlled trials is lacking. Moreover, the ethical implications of marketing skin-whitening treatments, particularly in regions with colorism-related social pressures, have prompted calls for stricter oversight and the development of culturally sensitive guidelines [153,176]. These controversies underscore the need for rigorous mechanistic studies, standardized clinical protocols, and transparent patient education.

As glutathione gains broader relevance in dermatology and regenerative medicine, its use should be guided by robust evidence, ethical considerations, and a nuanced understanding of its biological complexity.

### 4.4. Glutathione and Tumor Progression

Glutathione (GSH) plays a complex and pivotal role in tumor progression and cancer therapy resistance. It functions as a critical cellular antioxidant, regulating redox homeostasis, cell differentiation, proliferation, and apoptosis. Dysregulation of GSH levels is implicated in the etiology and advancement of many human cancers. Once malignant transformation has occurred, glutathione (GSH) often shifts from a protective antioxidant to a facilitator of tumor progression. Cancer cells experience high levels of oxidative stress due to rapid proliferation, metabolic rewiring, and mitochondrial dysfunction. To survive these conditions, many tumors upregulate GSH synthesis, recycling, and cystine import through the system Xc^−^ transporter. This enhanced redox buffering capacity enables cancer cells to maintain a reduced intracellular environment, supporting growth, survival, and metabolic flexibility [177,178,179].

Cancer cells often exhibit elevated GSH concentrations compared to normal tissues, which enhances their antioxidant capacity, allowing them to withstand oxidative stress that would otherwise limit tumor growth. Elevated GSH levels stabilize oncogenic signaling pathways, including those governing proliferation, survival, and epithelial–mesenchymal transition. By limiting ROS-induced damage, GSH allows tumor cells to tolerate genomic instability and sustain aggressive phenotypes. High GSH availability also promotes resistance to apoptosis by preserving mitochondrial membrane integrity and inhibiting redox-sensitive death pathways. A key component of this redox network is the GSH–GPX4 axis, which suppresses ferroptosis by preventing lipid peroxidation; this mechanism is increasingly recognized as essential for tumor cell survival and therapy resistance. By suppressing ferroptosis, this pathway allows cancer cells to survive under high oxidative stress and contributes to both chemoresistance and radioresistance. Evidence across multiple cancer models shows that modulation of GSH availability can alter susceptibility to ferroptotic cell death, raising concerns that systemic GSH supplementation might inadvertently support tumor progression in individuals with pre-existing or undiagnosed malignancies. These considerations underscore the need for a nuanced interpretation of glutathione biology in cancer and highlight the importance of carefully contextualizing redox-modulating interventions within oncologic risk frameworks [112,180,181].

A major consequence of this metabolic adaptation is resistance to anticancer therapies. Many chemotherapeutic agents and radiotherapy modalities rely on ROS generation or oxidative DNA damage to induce tumor cell death. Increased GSH neutralizes these reactive species, detoxifies electrophilic drug metabolites, and enhances DNA repair, thereby reducing treatment efficacy. This redox-driven resistance is further amplified by the upregulation of glutathione S-transferases, which conjugate GSH to cytotoxic compounds and facilitate their efflux. However, in many cancers, elevated glutathione levels confer resistance to chemotherapeutic agents, such as in breast, colon, lung, and bone marrow cancers. This dual role of GSH, as both protective and pathogenic, underscores the therapeutic potential of targeting glutathione metabolism to enhance the efficacy of cancer treatment. Because of this duality, glutathione represents both a vulnerability and a therapeutic target in oncology [182,183,184].

Understanding how glutathione metabolism is reprogrammed during tumor progression provides a conceptual framework for developing redox-based therapeutic interventions and for interpreting the paradoxical role of antioxidants in cancer biology.

Although none of the included dermatologic studies reported oncologic adverse events, the theoretical risk warrants caution, particularly in high-dose or long-term systemic use. Future research should address the safety profile of glutathione in populations at risk of malignancy and explore strategies to balance antioxidant benefits with potential oncologic implications.

### 4.5. Pharmacovigilance Gaps

Despite the growing use of glutathione across medical and esthetic settings, significant pharmacovigilance gaps limit the ability to accurately assess its long-term safety, real-world adverse event profile, and risk–benefit balance. These gaps arise from inconsistent reporting practices, heterogeneous product quality, and the widespread use of unregulated formulations, particularly in esthetic clinics.

#### 4.5.1. Underreporting of Adverse Events

Adverse reactions to glutathione—especially in intravenous administration—are likely underreported due to:

(1) Use in non-medical or minimally supervised esthetic settings;
(2) Lack of standardized monitoring protocols;
(3) Patient reluctance to report complications associated with cosmetic procedures;
(4) Absence of mandatory reporting systems in many countries.

This underreporting obscures the true incidence of hypersensitivity reactions, renal stress, thyroid disturbances, and other potential systemic effects [185,186,187,188,189].

#### 4.5.2. Variability in Product Quality and Composition

Pharmacovigilance is further complicated by:

(1) Inconsistent purity and concentration across commercial products;
(2) Presence of unverified or counterfeit injectable formulations;
(3) Lack of Good Manufacturing Practice (GMP) oversight in some regions;
(4) Undeclared additives or stabilizers that may contribute to toxicity.

Such variability makes it difficult to attribute adverse events to glutathione itself rather than to contaminants or formulation inconsistencies [190,191,192].

#### 4.5.3. Inadequate Regulatory Oversight

Regulatory agencies in several countries have issued warnings about unapproved injectable glutathione; however, enforcement remains inconsistent. These warnings often emphasize that injectable glutathione is not approved or authorized for cosmetic uses, such as skin lightening, and that its unregulated use may pose significant health risks. Such warnings highlight that injectable glutathione products marketed without regulatory approval may be counterfeit, substandard, or improperly manufactured, raising patient safety concerns. Esthetic clinics often operate outside medical regulatory frameworks, and cross-border online sales circumvent national safety controls. This regulatory fragmentation leads to uneven safety standards and limited traceability of adverse events. Globally, regulatory bodies continue to stress the importance of adhering to approved pharmaceutical standards to prevent the distribution and use of unapproved injectable glutathione formulations [193,194].

These regulatory measures are aimed at protecting public health by minimizing exposure to unsafe or ineffective glutathione products, especially for injectable and esthetic uses. To ensure patient safety and produce reliable long-term data, it is crucial to strengthen surveillance systems, standardize formulations and dosing protocols, and enforce regulatory oversight.

### 4.6. Limitations of the Present Review

Despite the growing interest in glutathione’s dermatological applications, current evidence is limited by short study durations, small sample sizes, and single-center designs. Most available trials span only 4 to 12 weeks and focus on narrow endpoints, such as the melanin index or subjective skin brightness, without assessing long-term safety, sustained efficacy, or histological changes. Moreover, variability in formulations, dosing regimens, and patient demographics complicates cross-study comparisons and hinders the development of standardized protocols. To address these gaps, longitudinal multicenter clinical trials are needed to evaluate the effects of glutathione over extended periods and across diverse populations. These trials are essential not only for validating glutathione’s therapeutic role in dermatology but also for guiding the evidence-based integration of glutathione into anti-aging, pigmentary, and regenerative protocols.

### 4.7. Challenges and Future Directions

Although glutathione shows a promising therapeutic profile, its clinical use in dermatology faces several challenges that warrant further investigation. A significant limitation is its inconsistent bioavailability, particularly in oral formulations, which are susceptible to enzymatic degradation and hepatic clearance. The lack of standardized dosing protocols across topical, oral, and injectable routes continues to pose challenges, thereby complicating comparisons and reproducibility in clinical research. Additionally, regulatory inconsistencies, particularly those related to intravenous use, raise concerns about safety, quality control, and ethical marketing practices in esthetic medicine. The lack of large-scale, long-term randomized trials further limits the ability to draw definitive conclusions regarding efficacy, optimal treatment duration, and sustained outcomes across diverse skin types and conditions.

Future research should focus on developing advanced delivery systems, such as microneedle patches, glutathione-loaded nanoparticles, and transdermal hydrogels, to enhance skin penetration and cellular uptake while minimizing systemic risks. The integration of glutathione with synergistic antioxidants, peptides, and growth factors offers a promising approach to developing personalized anti-aging and regenerative therapies. Furthermore, employing biomarker-driven methods to assess redox status and treatment response may improve patient selection and enhance the precision of therapeutic interventions. As interest in integrative dermatology grows, glutathione’s role is likely to expand beyond esthetic applications into areas such as wound healing, scar modulation, and inflammatory skin disorders, provided that future research rigorously addresses current gaps and is translationally relevant.

## 5. Conclusions

Glutathione plays a pivotal role in dermatological science, bridging fundamental redox biology and its clinical and esthetic applications. At the molecular level, it acts as a primary antioxidant, detoxifier, and redox regulator, preserving cellular integrity by interacting with key enzymes, including glutathione peroxidase, glutathione reductase, and glutathione transferase. Its impact extends to keratinocyte proliferation, fibroblast function, melanogenesis, and extracellular matrix remodeling, all of which are crucial for maintaining skin homeostasis, facilitating regeneration, and enhancing resilience against aging. Despite these promising outcomes, significant heterogeneity persists across study designs, formulations, dosages, and outcome measures, limiting the ability to draw definitive conclusions regarding optimal therapeutic protocols. Safety data for parenteral glutathione remain incomplete, particularly for long-term or repeated administration. Given the central role of redox pathways in cellular homeostasis and disease, systemic glutathione modulation should be interpreted with caution. Until more rigorous clinical trials are available, injectable and intravenous formulations cannot be recommended as evidence-based interventions for cutaneous aging or regenerative indications.

Challenges related to bioavailability, stability, and standardization remain central barriers to consistent clinical efficacy. Moreover, mechanistic uncertainties and the redox paradox associated with tumor biology highlight the need for cautious interpretation and rigorous long-term safety evaluation.

Future research should prioritize well-designed randomized controlled trials, standardized glutathione formulations, and validated dermatologic endpoints. Advances in delivery technologies, combined antioxidant strategies, and mechanistic studies integrating redox biology and regenerative medicine may further clarify glutathione’s therapeutic potential. Overall, while current evidence supports the dermatologic utility of glutathione, continued investigation is essential to establish evidence-based guidelines and ensure safe, effective, and standardized clinical use.

## Figures and Tables

**Table 1:** General Characteristics of the Clinical Studies.

Characteristics	Summary of Findings
Study designs	Randomized controlled trials, non-randomized interventional studies, observational studies, in vivo and in vitro experiments
**Populations**	Healthy volunteers, individuals with hyperpigmentation, photoaged skin, animal models, and cellular models
**Interventions**	Topical, oral, injectable glutathione; advanced delivery systems
**Duration of interventions**	2–12 weeks in clinical studies; variable in experimental studies
**Dermatologic outcomes assessed**	Pigmentation, brightness, hydration, elasticity, oxidative stress markers, and post-procedural recovery
**Anatomical sites**	Face, neck, forearm, and experimental tissue models

**Table 2:** Clinical Outcomes by Mode of Glutathione Administration.

Administration Route	Main Reported Outcomes	Typical Duration	Notes
Topical	Increased brightness, improved hydration, reduced localized pigmentation	2–12 weeks	Efficacy influenced by vehicle and formulation stability
Oral	Moderate reduction in overall pigmentation, improved antioxidant status	4–12 weeks	High variability in bioavailability
Injectable	Rapid elevation of systemic glutathione levels, improved tone uniformity	1–8 weeks	Fewer controlled studies are available
Advanced delivery systems	Enhanced stability, improved penetration, controlled release	Preclinical	Promising but limited clinical validation

## Data Availability

The original contributions presented in this study are included in the article and the Supplementary Materials.

## Supplementary Materials

The following supporting information can be downloaded at: https://www.mdpi.com/article/10.3390/molecules31060981/s1, Table S1: Characteristics of Included Studies; Table S2: Risk of Bias; and the PRISMA 2020 Checklist [195].

## References

[1] Ansary T.M., Hossain M.R., Kamiya K., Komine M., Ohtsuki M. (2021). Inflammatory Molecules Associated with Ultraviolet Radiation-Mediated Skin Aging. Int. J. Mol. Sci..

[2] Guerrero-Navarro L., Jansen-Dürr P., Cavinato M. (2024). Synergistic interplay of UV radiation and urban particulate matter induces impairment of autophagy and alters cellular fate in senescence-prone human dermal fibroblasts. Aging Cell.

[3] Soheilifar M.H., Masoudi-Khoram N., Shirkavand A., Ghorbanifar S. (2022). Non-coding RNAs in photoaging-related mechanisms: A new paradigm in skin health. Biogerontology.

[4] Fisher G.J., Kang S., Varani J., Bata-Csorgo Z., Wan Y., Datta S., Voorhees J.J. (2002). Mechanisms of photoaging and chronological skin aging. Arch. Dermatol..

[5] Parrado C., Mercado-Saenz S., Perez-Davo A., Gilaberte Y., Gonzalez S., Juarranz A. (2019). Environmental Stressors on Skin Aging. Mechanistic Insights. Front. Pharmacol..

[6] Bouayed J., Bohn T. (2010). Exogenous antioxidants—Double-edged swords in cellular redox state: Health beneficial effects at physiologic doses versus deleterious effects at high doses. Oxidative Med. Cell. Longev..

[7] Stanescu C., Chiscop I., Mihalache D., Popa F., Tamas C., Stoleriu G. (2025). Skin Aging and Carotenoids: A Systematic Review of Their Multifaceted Protective Mechanisms. Nutrients.

[8] Kim J., Gong Y.X., Jeong E.M. (2023). Measuring Glutathione Regeneration Capacity in Stem Cells. Int. J. Stem Cells.

[9] Labunskyy V.M., Gladyshev V.N. (2013). Role of reactive oxygen species-mediated signaling in aging. Antioxid. Redox Signal..

[10] Süntar I., Çetinkaya S., Panieri E., Saha S., Buttari B., Profumo E., Saso L. (2021). Regulatory Role of Nrf2 Signaling Pathway in Wound Healing Process. Molecules.

[11] Kalinina E.V., Chernov N.N., Novichkova M.D. (2014). Role of glutathione, glutathione transferase, and glutaredoxin in regulation of redox-dependent processes. Biochemistry.

[12] Hunt M., Torres M., Bachar-Wikstrom E., Wikstrom J.D. (2024). Cellular and molecular roles of reactive oxygen species in wound healing. Commun. Biol..

[13] Aquilano K., Baldelli S., Ciriolo M.R. (2014). Glutathione: New roles in redox signaling for an old antioxidant. Front. Pharmacol..

[14] Gasmi A., Nasreen A., Lenchyk L., Lysiuk R., Peana M., Shapovalova N., Piscopo S., Komisarenko M., Shanaida M., Smetanina K. (2024). An Update on Glutathione’s Biosynthesis, Metabolism, Functions, and Medicinal Purposes. Curr. Med. Chem..

[15] Berndt C., Lillig C.H. (2017). Glutathione, Glutaredoxins, and Iron. Antioxid. Redox Signal..

[16] Riskowski R.A., Nemeth R.S., Borgognoni K., Ackerson C.J. (2019). Enzyme-Catalyzed in situ Synthesis of Temporally and Spatially Distinct CdSe Quantum Dots in Biological Backgrounds. J. Phys. Chem. C Nanomater. Interfaces.

[17] Scirè A., Cianfruglia L., Minnelli C., Bartolini D., Torquato P., Principato G., Galli F., Armeni T. (2019). Glutathione compartmentalization and its role in glutathionylation and other regulatory processes of cellular pathways. BioFactors.

[18] Cacciatore I., Cornacchia C., Pinnen F., Mollica A., Di Stefano A. (2010). Prodrug approach for increasing cellular glutathione levels. Molecules.

[19] Vázquez-Meza H., Vilchis-Landeros M.M., Vázquez-Carrada M., Uribe-Ramírez D., Matuz-Mares D. (2023). Cellular Compartmentalization, Glutathione Transport and Its Relevance in Some Pathologies. Antioxidants.

[20] Diaz-Vivancos P., de Simone A., Kiddle G., Foyer C.H. (2015). Glutathione—Linking cell proliferation to oxidative stress. Free Radic. Biol. Med..

[21] Chakraborty S., Sircar E., Bhattacharyya C., Choudhuri A., Mishra A., Dutta S., Bhatta S., Sachin K., Sengupta R. (2022). S-Denitrosylation: A Crosstalk between Glutathione and Redoxin Systems. Antioxidants.

[22] Bettendorff L. (2022). Reduced Nucleotides, Thiols and O_2_ in Cellular Redox Balance: A Biochemist’s View. Antioxidants.

[23] Maestri E., Duszka K., Kuznetsov V.A. (2021). Immunity Depletion, Telomere Imbalance, and Cancer-Associated Metabolism Pathway Aberrations in Intestinal Mucosa upon Short-Term Caloric Restriction. Cancers.

[24] Narayanankutty A., Job J.T., Narayanankutty V. (2019). Glutathione, an Antioxidant Tripeptide: Dual Roles in Carcinogenesis and Chemoprevention. Curr. Protein Pept. Sci..

[25] Dergousova E.A., Petrushanko I.Y., Klimanova E.A., Mitkevich V.A., Ziganshin R.H., Lopina O.D., Makarov A.A. (2017). Effect of Reduction of Redox Modifications of Cys-Residues in the Na,K-ATPase α1-Subunit on Its Activity. Biomolecules.

[26] Mazari A.M.A., Zhang L., Ye Z.W., Zhang J., Tew K.D., Townsend D.M. (2023). The Multifaceted Role of Glutathione S-Transferases in Health and Disease. Biomolecules.

[27] Anashkina A.A., Simonenko S.Y., Orlov Y.L., Petrushanko I.Y. (2023). Glutathione Non-Covalent Binding Sites on Hemoglobin and Major Glutathionylation Target betaCys93 Are Conservative among Both Hypoxia-Sensitive and Hypoxia-Tolerant Mammal Species. Int. J. Mol. Sci..

[28] Li W., Busu C., Circu M.L., Aw T.Y. (2012). Glutathione in cerebral microvascular endothelial biology and pathobiology: Implications for brain homeostasis. Int. J. Cell Biol..

[29] Kim K., Choi J., Iram S., Kim J. (2024). Regulation of Glutathione *S*-Transferase Omega 1 Mediated by Cysteine Residues Sensing the Redox Environment. Int. J. Mol. Sci..

[30] Lubos E., Loscalzo J., Handy D.E. (2011). Glutathione peroxidase-1 in health and disease: From molecular mechanisms to therapeutic opportunities. Antioxid. Redox Signal..

[31] Chang C., Worley B.L., Phaëton R., Hempel N. (2020). Extracellular Glutathione Peroxidase GPx3 and Its Role in Cancer. Cancers.

[32] Handy D.E., Lubos E., Yang Y., Galbraith J.D., Kelly N., Zhang Y.Y., Leopold J.A., Loscalzo J. (2009). Glutathione peroxidase-1 regulates mitochondrial function to modulate redox-dependent cellular responses. J. Biol. Chem..

[33] Sands K.N., Tuck T.A., Back T.G. (2018). Cyclic Seleninate Esters, Spirodioxyselenuranes and Related Compounds: New Classes of Biological Antioxidants That Emulate Glutathione Peroxidase. Chemistry.

[34] Vašková J., Kočan L., Vaško L., Perjési P. (2023). Glutathione-Related Enzymes and Proteins: A Review. Molecules.

[35] Lushchak V.I. (2012). Glutathione homeostasis and functions: Potential targets for medical interventions. J. Amino Acids.

[36] Aoyama K., Nakaki T. (2015). Glutathione in Cellular Redox Homeostasis: Association with the Excitatory Amino Acid Carrier 1 (EAAC1). Molecules.

[37] Chai Y.C., Mieyal J.J. (2023). Glutathione and Glutaredoxin-Key Players in Cellular Redox Homeostasis and Signaling. Antioxidants.

[38] Ooi B.K., Goh B.H., Yap W.H. (2017). Oxidative Stress in Cardiovascular Diseases: Involvement of Nrf2 Antioxidant Redox Signaling in Macrophage Foam Cells Formation. Int. J. Mol. Sci..

[39] Dong S.C., Sha H.H., Xu X.Y., Hu T.M., Lou R., Li H., Wu J.Z., Dan C., Feng J. (2018). Glutathione *S*-transferase π: A potential role in antitumor therapy. Drug Des. Dev. Ther..

[40] Dringen R., Brandmann M., Hohnholt M.C., Blumrich E.M. (2015). Glutathione-Dependent Detoxification Processes in Astrocytes. Neurochem. Res..

[41] Hong H., Lu Y., Ji Z.N., Liu G.Q. (2006). Up-regulation of P-glycoprotein expression by glutathione depletion-induced oxidative stress in rat brain microvessel endothelial cells. J. Neurochem..

[42] Presnell C.E., Bhatti G., Numan L.S., Lerche M., Alkhateeb S.K., Ghalib M., Shammaa M., Kavdia M. (2013). Computational insights into the role of glutathione in oxidative stress. Curr. Neurovascular Res..

[43] Hushcha Y., Blo I., Oton-Gonzalez L., Mauro G.D., Martini F., Tognon M., Mattei M. (2021). microRNAs in the Regulation of Melanogenesis. Int. J. Mol. Sci..

[44] Kondo T., Hearing V.J. (2011). Update on the regulation of mammalian melanocyte function and skin pigmentation. Expert Rev. Dermatol..

[45] Putri S.A., Maharani R., Maksum I.P., Siahaan T.J. (2025). Peptide Design for Enhanced Anti-Melanogenesis: Optimizing Molecular Weight, Polarity, and Cyclization. Drug Des. Dev. Ther..

[46] Jeon G., Kim C., Cho U.M., Hwang E.T., Hwang H.S., Min J. (2021). Melanin-Decolorizing Activity of Antioxidant Enzymes, Glutathione Peroxidase, Thiol Peroxidase, and Catalase. Mol. Biotechnol..

[47] Pillaiyar T., Namasivayam V., Manickam M., Jung S.H. (2018). Inhibitors of Melanogenesis: An Updated Review. J. Med. Chem..

[48] Qian W., Liu W., Zhu D., Cao Y., Tang A., Gong G., Su H. (2020). Natural skin-whitening compounds for the treatment of melanogenesis (Review). Exp. Ther. Med..

[49] Schmidt A., von Woedtke T., Vollmar B., Hasse S., Bekeschus S. (2019). Nrf2 signaling and inflammation are key events in physical plasma-spurred wound healing. Theranostics.

[50] Leavitt T., Hu M.S., Marshall C.D., Barnes L.A., Lorenz H.P., Longaker M.T. (2016). Scarless wound healing: Finding the right cells and signals. Cell Tissue Res..

[51] Stanescu C., Chiscop I., Mihalache D., Boev M., Tamas C., Stoleriu G. (2025). The Roles of Micronutrition and Nutraceuticals in Enhancing Wound Healing and Tissue Regeneration: A Systematic Review. Molecules.

[52] Lekas M., Lekas P., Latter D.A., Kutryk M.B., Stewart D.J. (2006). Growth factor-induced therapeutic neovascularization for ischaemic vascular disease: Time for a re-evaluation?. Curr. Opin. Cardiol..

[53] Ahn A., Frishman W.H., Gutwein A., Passeri J., Nelson M. (2008). Therapeutic angiogenesis: A new treatment approach for ischemic heart disease—Part II. Cardiol. Rev..

[54] Pessoa A.F., Florim J.C., Rodrigues H.G., Andrade-Oliveira V., Teixeira S.A., Vitzel K.F., Curi R., Saraiva Câmara N.O., Muscará M.N., Lamers M.L. (2016). Oral administration of antioxidants improves skin wound healing in diabetic mice. Wound Repair Regen..

[55] Bains V.K., Bains R. (2015). The antioxidant master glutathione and periodontal health. Dent. Res. J..

[56] Arnal-Forné M., Borrás C. (2025). Therapies targeting redox balance improve wound healing: A systematic review and meta-analysis. Redox Med..

[57] Lin X., Lai Y. (2024). Scarring Skin: Mechanisms and Therapies. Int. J. Mol. Sci..

[58] Sangha M.S., Deroide F., Meys R. (2024). Wound healing, scarring and management. Clin. Exp. Dermatol..

[59] Stanescu C., Anghel L., Tamas C., Ciubara A. (2025). Education of Patients and Their Families to Manage Emotional Impact of Skin Scars. BRAIN. Broad Res. Artif. Intell. Neurosci..

[60] Giustarini D., Milzani A., Dalle-Donne I., Rossi R. (2023). How to Increase Cellular Glutathione. Antioxidants.

[61] Świderska-Kołacz G., Jefimow M., Klusek J., Rączka N., Zmorzyński S., Wojciechowska A., Stanisławska I., Łyp M., Czerwik-Marcinkowska J. (2021). Influence of Algae Supplementation on the Concentration of Glutathione and the Activity of Glutathione Enzymes in the Mice Liver and Kidney. Nutrients.

[62] Al-Temimi A.A., Al-Mossawi A.E., Al-Hilifi S.A., Korma S.A., Esatbeyoglu T., Rocha J.M., Agarwal V. (2023). Glutathione for Food and Health Applications with Emphasis on Extraction, Identification, and Quantification Methods: A Review. Metabolites.

[63] Minich D.M., Brown B.I. (2019). A Review of Dietary (Phyto)Nutrients for Glutathione Support. Nutrients.

[64] Liebman S.E., Le T.H. (2021). Eat Your Broccoli: Oxidative Stress, NRF2, and Sulforaphane in Chronic Kidney Disease. Nutrients.

[65] Nogales F., Ojeda M.L., Fenutría M., Murillo M.L., Carreras O. (2013). Role of selenium and glutathione peroxidase on development, growth, and oxidative balance in rat offspring. Reproduction.

[66] Dogan H., Coteli E., Karatas F. (2016). Determination of Glutathione, Selenium, and Malondialdehyde in Different Edible Mushroom Species. Biol. Trace Elem. Res..

[67] Baldelli S., Ciccarone F., Limongi D., Checconi P., Palamara A.T., Ciriolo M.R. (2019). Glutathione and Nitric Oxide: Key Team Players in Use and Disuse of Skeletal Muscle. Nutrients.

[68] Zhou X.J., Vaziri N.D., Wang X.Q., Silva F.G., Laszik Z. (2002). Nitric oxide synthase expression in hypertension induced by inhibition of glutathione synthase. J. Pharmacol. Exp. Ther..

[69] Chen X.X., Niu L.Y., Yang Q.Z. (2021). Visualizing the Underlying Signaling Pathway Related to Nitric Oxide and Glutathione in Cardiovascular Disease Therapy by a Sequentially Activated Fluorescent Probe. Anal. Chem..

[70] Niki E. (2011). Do free radicals play causal role in atherosclerosis? Low density lipoprotein oxidation and vitamin E revisited. J. Clin. Biochem. Nutr..

[71] Victor V.M., Rocha M., Solá E., Bañuls C., Garcia-Malpartida K., Hernández-Mijares A. (2009). Oxidative stress, endothelial dysfunction and atherosclerosis. Curr. Pharm. Des..

[72] Roşian Ş.H., Boarescu I., Boarescu P.M. (2025). Antioxidant and Anti-Inflammatory Effects of Bioactive Compounds in Atherosclerosis. Int. J. Mol. Sci..

[73] He X., Deng J., Yu X.J., Yang S., Yang Y., Zang W.J. (2020). Activation of M3AChR (Type 3 Muscarinic Acetylcholine Receptor) and Nrf2 (Nuclear Factor Erythroid 2-Related Factor 2) Signaling by Choline Alleviates Vascular Smooth Muscle Cell Phenotypic Switching and Vascular Remodeling. Arterioscler. Thromb. Vasc. Biol..

[74] Matsumori A. (2023). Nuclear Factor-κB is a Prime Candidate for the Diagnosis and Control of Inflammatory Cardiovascular Disease. Eur. Cardiol. Rev..

[75] Baines C.P. (2009). The mitochondrial permeability transition pore and ischemia-reperfusion injury. Basic Res. Cardiol..

[76] Kahl A., Stepanova A., Konrad C., Anderson C., Manfredi G., Zhou P., Iadecola C., Galkin A. (2018). Critical Role of Flavin and Glutathione in Complex I-Mediated Bioenergetic Failure in Brain Ischemia/Reperfusion Injury. Stroke.

[77] Hu Q., Zhou Q., Wu J., Wu X., Ren J. (2019). The Role of Mitochondrial DNA in the Development of Ischemia Reperfusion Injury. Shock.

[78] Jassem W., Fuggle S.V., Rela M., Koo D.D., Heaton N.D. (2002). The role of mitochondria in ischemia/reperfusion injury. Transplantation.

[79] Rashdan N.A., Shrestha B., Pattillo C.B. (2020). S-glutathionylation, friend or foe in cardiovascular health and disease. Redox Biol..

[80] Rozanski G.J., Xu Z. (2002). Glutathione and K(+) channel remodeling in postinfarction rat heart. Am. J. Physiol. Heart Circ. Physiol..

[81] Myszor I.T., Gudmundsson G.H. (2023). Modulation of innate immunity in airway epithelium for host-directed therapy. Front. Immunol..

[82] Vareille M., Kieninger E., Edwards M.R., Regamey N. (2011). The airway epithelium: Soldier in the fight against respiratory viruses. Clin. Microbiol. Rev..

[83] Chen T.H., Wang H.C., Chang C.J., Lee S.Y. (2024). Mitochondrial Glutathione in Cellular Redox Homeostasis and Disease Manifestation. Int. J. Mol. Sci..

[84] Cipollina C., Bruno A., Fasola S., Cristaldi M., Patella B., Inguanta R., Vilasi A., Aiello G., La Grutta S., Torino C. (2022). Cellular and Molecular Signatures of Oxidative Stress in Bronchial Epithelial Cell Models Injured by Cigarette Smoke Extract. Int. J. Mol. Sci..

[85] Koike Y., Hisada T., Utsugi M., Ishizuka T., Shimizu Y., Ono A., Murata Y., Hamuro J., Mori M., Dobashi K. (2007). Glutathione redox regulates airway hyperresponsiveness and airway inflammation in mice. Am. J. Respir. Cell Mol. Biol..

[86] Pekovic-Vaughan V., Gibbs J., Yoshitane H., Yang N., Pathiranage D., Guo B., Sagami A., Taguchi K., Bechtold D., Loudon A. (2014). The circadian clock regulates rhythmic activation of the NRF2/glutathione-mediated antioxidant defense pathway to modulate pulmonary fibrosis. Genes Dev..

[87] Zuo L., Wijegunawardana D. (2021). Redox Role of ROS and Inflammation in Pulmonary Diseases. Adv. Exp. Med. Biol..

[88] Reddy S.P. (2008). The antioxidant response element and oxidative stress modifiers in airway diseases. Curr. Mol. Med..

[89] van de Wetering C., Elko E., Berg M., Schiffers C.H.J., Stylianidis V., van den Berge M., Nawijn M.C., Wouters E.F.M., Janssen-Heininger Y.M.W., Reynaert N.L. (2021). Glutathione S-transferases and their implications in the lung diseases asthma and chronic obstructive pulmonary disease: Early life susceptibility?. Redox Biol..

[90] Tasaka S., Amaya F., Hashimoto S., Ishizaka A. (2008). Roles of oxidants and redox signaling in the pathogenesis of acute respiratory distress syndrome. Antioxid. Redox Signal..

[91] Di Tommaso N., Gasbarrini A., Ponziani F.R. (2021). Intestinal Barrier in Human Health and Disease. Int. J. Environ. Res. Public Health.

[92] Young V.B. (2012). The intestinal microbiota in health and disease. Curr. Opin. Gastroenterol..

[93] Sulaiman Y., Pacauskienė I.M., Šadzevičienė R., Anuzyte R. (2024). Oral and Gut Microbiota Dysbiosis Due to Periodontitis: Systemic Implications and Links to Gastrointestinal Cancer: A Narrative Review. Medicina.

[94] Yu X., Cheng L., Yi X., Li B., Li X., Liu X., Liu Z., Kong X. (2024). Gut phageome: Challenges in research and impact on human microbiota. Front. Microbiol..

[95] Xiang X., Wang H., Zhou W., Wang C., Guan P., Xu G., Zhao Q., He L., Yin Y., Li T. (2022). Glutathione Protects against Paraquat-Induced Oxidative Stress by Regulating Intestinal Barrier, Antioxidant Capacity, and CAR Signaling Pathway in Weaned Piglets. Nutrients.

[96] Shang Y., Siow Y.L., Isaak C.K., O K. (2016). Downregulation of Glutathione Biosynthesis Contributes to Oxidative Stress and Liver Dysfunction in Acute Kidney Injury. Oxidative Med. Cell. Longev..

[97] Bonetti L., Horkova V., Longworth J., Guerra L., Kurniawan H., Franchina D.G., Soriano-Baguet L., Grusdat M., Spath S., Koncina E. (2024). A Th17 cell-intrinsic glutathione/mitochondrial-IL-22 axis protects against intestinal inflammation. Cell Metab..

[98] Wolozin B., Behl C. (2000). Mechanisms of neurodegenerative disorders: Part 1: Protein aggregates. Arch. Neurol..

[99] Ren X., Zou L., Zhang X., Branco V., Wang J., Carvalho C., Holmgren A., Lu J. (2017). Redox Signaling Mediated by Thioredoxin and Glutathione Systems in the Central Nervous System. Antioxid. Redox Signal..

[100] Sabens Liedhegner E.A., Gao X.H., Mieyal J.J. (2012). Mechanisms of altered redox regulation in neurodegenerative diseases—Focus on S-glutathionylation. Antioxid. Redox Signal..

[101] Lana J.V., Rios A., Takeyama R., Santos N., Pires L., Santos G.S., Rodrigues I.J., Jeyaraman M., Purita J., Lana J.F. (2024). Nebulized Glutathione as a Key Antioxidant for the Treatment of Oxidative Stress in Neurodegenerative Conditions. Nutrients.

[102] Carvalho A.N., Lim J.L., Nijland P.G., Witte M.E., Van Horssen J. (2014). Glutathione in multiple sclerosis: More than just an antioxidant?. Mult. Scler..

[103] Liu H., Wang H., Shenvi S., Hagen T.M., Liu R.M. (2004). Glutathione metabolism during aging and in Alzheimer disease. Ann. N. Y. Acad. Sci..

[104] Chełchowska M., Gajewska J., Szczepanik E., Mazur J., Cychol A., Kuźniar-Pałka A., Ambroszkiewicz J. (2025). Oxidative Stress Indicated by Nuclear Transcription Factor Nrf2 and Glutathione Status in the Blood of Young Children with Autism Spectrum Disorder: Pilot Study. Antioxidants.

[105] Garcia-Bonilla L., Benakis C., Moore J., Iadecola C., Anrather J. (2014). Immune mechanisms in cerebral ischemic tolerance. Front. Neurosci..

[106] Aoyama K., Nakaki T. (2013). Impaired glutathione synthesis in neurodegeneration. Int. J. Mol. Sci..

[107] Elda Valenti G., Tasso B., Traverso N., Domenicotti C., Marengo B. (2023). Glutathione in cancer progression and chemoresistance: An update. Redox Exp. Med..

[108] Kennedy L., Sandhu J.K., Harper M.E., Cuperlovic-Culf M. (2020). Role of Glutathione in Cancer: From Mechanisms to Therapies. Biomolecules.

[109] Tamas C., Jemnoschi Hreniuc I.M., Tecuceanu A., Ciuntu B.M., Ibanescu C.L., Tamas I., Ianole V., Stanescu C., Pintilie C.T., Zamfir C.L. (2021). Non-Melanoma Facial Skin Tumors—The Correspondence between Clinical and Histological Diagnosis. Appl. Sci..

[110] Traverso N., Ricciarelli R., Nitti M., Marengo B., Furfaro A.L., Pronzato M.A., Marinari U.M., Domenicotti C. (2013). Role of glutathione in cancer progression and chemoresistance. Oxidative Med. Cell. Longev..

[111] Bansal A., Simon M.C. (2018). Glutathione metabolism in cancer progression and treatment resistance. J. Cell Biol..

[112] Sekhar K.R., Hanna D.N., Cyr S., Baechle J.J., Kuravi S., Balusu R., Rathmell K., Baregamian N. (2022). Glutathione peroxidase 4 inhibition induces ferroptosis and mTOR pathway suppression in thyroid cancer. Sci. Rep..

[113] Gronau S., Koenig-Greger D., Jerg M., Riechelmann H. (2003). GSTM1 enzyme concentration and enzyme activity in correlation to the genotype of detoxification enzymes in squamous cell carcinoma of the oral cavity. Oral Dis..

[114] Hu Q., Li C., Huang Y., Wei Z., Chen L., Luo Y., Li X. (2024). Effects of Glutathione S-Transferases (GSTM1, GSTT1 and GSTP1) gene variants in combination with smoking or drinking on cancers: A meta-analysis. Medicine.

[115] Li W., Li M., Qi J. (2021). Nano-Drug Design Based on the Physiological Properties of Glutathione. Molecules.

[116] Hash M.G., Forsyth A., Coleman B.A., Li V., Vinagolu-Baur J., Frasier K.M. (2025). Artificial Intelligence in the Evolution of Customized Skincare Regimens. Cureus.

[117] Frasier K., Li V., Sobotka M., Vinagolu-Baur J., Herrick G. (2024). The role of wearable technology in real-time skin health monitoring. JEADV Clin. Pract..

[118] Singh H., Nim K., Randhawa A.S., Ahluwalia S. (2024). Integrating clinical pharmacology and artificial intelligence: Potential benefits, challenges, and role of clinical pharmacologists. Expert Rev. Clin. Pharmacol..

[119] Elder A., Ring C., Heitmiller K., Gabriel Z., Saedi N. (2021). The role of artificial intelligence in cosmetic dermatology-Current, upcoming, and future trends. J. Cosmet. Dermatol..

[120] Nahm W.J., Nikas C., Goldust M., Horneck N., Cervantes J.A., Burshtein J., Tsoukas M. (2025). Exosomes in Dermatology: A Comprehensive Review of Current Applications, Clinical Evidence, and Future Directions. Int. J. Dermatol..

[121] Torres A., Almeida I.F., Oliveira R. (2024). An Overview of Proprietary Vehicles/Bases for Topical Compounding Medicines and Cosmetics. Cosmetics.

[122] Mayba J.N., Gooderham M.J. (2018). A Guide to Topical Vehicle Formulations. J. Cutan. Med. Surg..

[123] Buonocore D., Grosini M., Giardina S., Michelotti A., Carrabetta M., Seneci A., Verri M., Dossena M., Marzatico F. (2016). Bioavailability Study of an Innovative Orobuccal Formulation of Glutathione. Oxidative Med. Cell. Longev..

[124] Watanabe F., Hashizume E., Chan G.P., Kamimura A. (2014). Skin-whitening and skin-condition-improving effects of topical oxidized glutathione: A double-blind and placebo-controlled clinical trial in healthy women. Clin. Cosmet. Investig. Dermatol..

[125] Grandi V., Milanesi N., Sessa M., Gola M., Cappugi P., Pimpinelli N. (2019). Efficacy and safety of S-acyl glutathione 2% cream vs. placebo against UVB-induced erythema: A randomized, double-blinded clinical trial. G. Ital. Dermatol. Venereol..

[126] Cui X., Mi T., Xiao X., Zhang H., Dong Y., Huang N., Gao P., Lee J., Guelakis M., Gu X. (2024). Topical glutathione amino acid precursors protect skin against environmental and oxidative stress. J. Eur. Acad. Dermatol. Venereol..

[127] Arjinpathana N., Asawanonda P. (2012). Glutathione as an oral whitening agent: A randomized, double-blind, placebo-controlled study. J. Dermatol. Treat..

[128] Handog E.B., Datuin M.S., Singzon I.A. (2016). An open-label, single-arm trial of the safety and efficacy of a novel preparation of glutathione as a skin-lightening agent in Filipino women. Int. J. Dermatol..

[129] Duperray J., Sergheraert R., Chalothorn K., Tachalerdmanee P., Perin F. (2022). The effects of the oral supplementation of L-Cystine associated with reduced L-Glutathione-GSH on human skin pigmentation: A randomized, double-blinded, benchmark- and placebo-controlled clinical trial. J. Cosmet. Dermatol..

[130] Richie J.P., Nichenametla S., Neidig W., Calcagnotto A., Haley J.S., Schell T.D., Muscat J.E. (2015). Randomized controlled trial of oral glutathione supplementation on body stores of glutathione. Eur. J. Nutr..

[131] Weschawalit S., Thongthip S., Phutrakool P., Asawanonda P. (2017). Glutathione and its antiaging and antimelanogenic effects. Clin. Cosmet. Investig. Dermatol..

[132] Sonthalia S., Daulatabad D., Sarkar R. (2016). Glutathione as a skin whitening agent: Facts, myths, evidence and controversies. Indian J. Dermatol. Venereol. Leprol..

[133] Dilokthornsakul W., Dhippayom T., Dilokthornsakul P. (2019). The clinical effect of glutathione on skin color and other related skin conditions: A systematic review. J. Cosmet. Dermatol..

[134] Sarkar R., Yadav V., Yadav T.P.J., Mandal I. (2025). Glutathione as a skin-lightening agent and in melasma: A systematic review. Int. J. Dermatol..

[135] Johnstone T., Quinn E., Tobin S., Davis R., Najjar Z., Battye B., Gupta L. (2018). Seven cases of probable endotoxin poisoning related to contaminated glutathione infusions. Epidemiol. Infect..

[136] Guilherme V.A., Ribeiro L.N.M., Tofoli G.R., Franz-Montan M., de Paula E., de Jesus M.B. (2017). Current Challenges and Future of Lipid nanoparticles formulations for topical drug application to oral mucosa, skin, and eye. Curr. Pharm. Des..

[137] Wu S., Liu G., Shao P., Lin X., Yu J., Chen H., Li H., Feng S. (2024). Transdermal Sustained Release Properties and Anti-Photoaging Efficacy of Liposome-Thermosensitive Hydrogel System. Adv. Healthc. Mater..

[138] Patel D., Patel B., Thakkar H. (2021). Lipid Based Nanocarriers: Promising Drug Delivery System for Topical Application. Eur. J. Lipid Sci. Technol..

[139] Benson H.A. (2017). Elastic Liposomes for Topical and Transdermal Drug Delivery. Methods Mol. Biol..

[140] Carita A.C., Eloy J.O., Chorilli M., Lee R.J., Leonardi G.R. (2018). Recent Advances and Perspectives in Liposomes for Cutaneous Drug Delivery. Curr. Med. Chem..

[141] Dinh L., Hwang S.J., Yan B. (2025). Hydrogel Conjugation: Engineering of Hydrogels for Drug Delivery. Pharmaceutics.

[142] Gao Z., Golland B., Tronci G., Thornton P.D. (2019). A redox-responsive hyaluronic acid-based hydrogel for chronic wound management. J. Mater. Chem. B.

[143] Li Z., Liu J., Song J., Yin Z., Zhou F., Shen H., Wang G., Su J. (2024). Multifunctional hydrogel-based engineered extracellular vesicles delivery for complicated wound healing. Theranostics.

[144] Zhu B., Zong T., Zheng R., Chen X., Zhou Y., Liu Y., Yan J., Zhao B., Yin J. (2024). Acid and Glutathione Dual-Responsive, Injectable and Self-Healing Hydrogels for Controlled Drug Delivery. Biomacromolecules.

[145] Liu M., Sharma M., Lu G., Zhang Z., Song W., Wen J. (2025). Innovative Solid Lipid Nanoparticle-Enriched Hydrogels for Enhanced Topical Delivery of L-Glutathione: A Novel Approach to Anti-Ageing. Pharmaceutics.

[146] Santacroce G., Gentile A., Soriano S., Novelli A., Lenti M.V., Di Sabatino A. (2023). Glutathione: Pharmacological aspects and implications for clinical use in non-alcoholic fatty liver disease. Front. Med..

[147] Dawi J., Misakyan Y., Affa S., Kades S., Narasimhan A., Hajjar F., Besser M., Tumanyan K., Venketaraman V. (2025). Oxidative Stress, Glutathione Insufficiency, and Inflammatory Pathways in Type 2 Diabetes Mellitus: Implications for Therapeutic Interventions. Biomedicines.

[148] Balendiran G.K., Dabur R., Fraser D. (2004). The role of glutathione in cancer. Cell Biochem. Funct..

[149] Desideri E., Ciccarone F., Ciriolo M.R. (2019). Targeting Glutathione Metabolism: Partner in Crime in Anticancer Therapy. Nutrients.

[150] Zhang D., Luo G., Jin K., Bao X., Huang L., Ke J. (2023). The underlying mechanisms of cisplatin-induced nephrotoxicity and its therapeutic intervention using natural compounds. Naunyn-Schmiedeberg’s Arch. Pharmacol..

[151] Casanova A.G., Hernández-Sánchez M.T., López-Hernández F.J., Martínez-Salgado C., Prieto M., Vicente-Vicente L., Morales A.I. (2020). Systematic review and meta-analysis of the efficacy of clinically tested protectants of cisplatin nephrotoxicity. Eur. J. Clin. Pharmacol..

[152] Yang Z., Lyu J., Qian J., Wang Y., Liu Z., Yao Q., Chen T., Cao Y., Xie J. (2025). Glutathione: A naturally occurring tripeptide for functional metal nanomaterials. Chem. Sci..

[153] Ramirez S., Cullen C., Ahdoot R., Scherz G. (2024). The Primacy of Ethics in Aesthetic Medicine: A Review. Plast. Reconstr. Surg. Glob. Open.

[154] da Prato E.B., Cartier H., Margara A., Molina B., Tateo A., Grimolizzi F., Spagnolo A.G. (2024). The ethical foundations of patient-centered care in aesthetic medicine. Philos. Ethics Humanit. Med..

[155] Waly M.I., Al-Attabi Z., Guizani N. (2015). Low Nourishment of Vitamin C Induces Glutathione Depletion and Oxidative Stress in Healthy Young Adults. Prev. Nutr. Food Sci..

[156] Tram N.K., McLean R.M., Swindle-Reilly K.E. (2021). Glutathione Improves the Antioxidant Activity of Vitamin C in Human Lens and Retinal Epithelial Cells: Implications for Vitreous Substitutes. Curr. Eye Res..

[157] Gao F., Yao C.L., Gao E., Mo Q.Z., Yan W.L., McLaughlin R., Lopez B.L., Christopher T.A., Ma X.L. (2002). Enhancement of glutathione cardioprotection by ascorbic acid in myocardial reperfusion injury. J. Pharmacol. Exp. Ther..

[158] Guaiquil V.H., Vera J.C., Golde D.W. (2001). Mechanism of vitamin C inhibition of cell death induced by oxidative stress in glutathione-depleted HL-60 cells. J. Biol. Chem..

[159] Khushi Kapoor S.S., Rukaiah Fatma Begum A.S.S., Afreen N. (2025). Exploring Niacinamide as a Multifunctional Agent for Skin Health and Rejuvenation. Curr. Pharm. Biotechnol..

[160] Gehring W. (2004). Nicotinic acid/niacinamide and the skin. J. Cosmet. Dermatol..

[161] Rolfe H.M. (2014). A review of nicotinamide: Treatment of skin diseases and potential side effects. J. Cosmet. Dermatol..

[162] Serre C., Busuttil V., Botto J.M. (2018). Intrinsic and extrinsic regulation of human skin melanogenesis and pigmentation. Int. J. Cosmet. Sci..

[163] Hakozaki T., Takiwaki H., Miyamoto K., Sato Y., Arase S. (2006). Ultrasound enhanced skin-lightening effect of vitamin C and niacinamide. Skin. Res. Technol..

[164] Foyer C.H. (2001). Prospects for enhancement of the soluble antioxidants, ascorbate and glutathione. Biofactors.

[165] Colangelo M.T., Galli C., Guizzardi S. (2020). The effects of polydeoxyribonucleotide on wound healing and tissue regeneration: A systematic review of the literature. Regen. Med..

[166] Jeong W., Yang C.E., Roh T.S., Kim J.H., Lee J.H., Lee W.J. (2017). Scar Prevention and Enhanced Wound Healing Induced by Polydeoxyribonucleotide in a Rat Incisional Wound-Healing Model. Int. J. Mol. Sci..

[167] Khan A., Wang G., Zhou F., Gong L., Zhang J., Qi L., Cui H. (2022). Polydeoxyribonucleotide: A promising skin anti-aging agent. Chin. J. Plast. Reconstr. Surg..

[168] Park H.J., Byun K.-A., Oh S., Kim H.M., Chung M.S., Son K.H., Byun K. (2022). The Combination of Niacinamide, Vitamin C, and PDRN Mitigates Melanogenesis by Modulating Nicotinamide Nucleotide Transhydrogenase. Molecules.

[169] Wahab S., Anwar A.I., Zainuddin A.N., Hutabarat E.N., Anwar A.A., Kurniadi I. (2021). Combination of topical and oral glutathione as a skin-whitening agent: A double-blind randomized controlled clinical trial. Int. J. Dermatol..

[170] Schmitt B., Vicenzi M., Garrel C., Denis F.M. (2015). Effects of N-acetylcysteine, oral glutathione (GSH) and a novel sublingual form of GSH on oxidative stress markers: A comparative crossover study. Redox Biol..

[171] Yin N., Harris P.W.R., Liu M., Sun J., Chen G., We J., Brimble M.A. (2025). Enhancing the Oral Bioavailability of Glutathione Using Innovative Analogue Approaches. Pharmaceutics.

[172] Dehkordi H.T., Ghasemi S. (2024). Glutathione Therapy in Diseases: Challenges and Potential Solutions for Therapeutic Advancement. Curr. Mol. Med..

[173] Shen H., Wang W. (2021). Effect of glutathione liposomes on diabetic nephropathy based on oxidative stress and polyol pathway mechanism. J. Liposome Res..

[174] Xia Q., Saupe A., Müller R.H., Souto E.B. (2007). Nanostructured lipid carriers as novel carrier for sunscreen formulations. Int. J. Cosmet. Sci..

[175] Dobreva M., Stefanov S., Andonova V. (2020). Natural Lipids as Structural Components of Solid Lipid Nanoparticles and Nanostructured Lipid Carriers for Topical Delivery. Curr. Pharm. Des..

[176] Villarama C.D., Maibach H.I. (2005). Glutathione as a depigmenting agent: An overview. Int. J. Cosmet. Sci..

[177] Estrela J.M., Ortega A., Obrador E. (2006). Glutathione in cancer biology and therapy. Crit. Rev. Clin. Lab. Sci..

[178] Marengo B., Pulliero A., Izzotti A., Domenicotti C. (2020). miRNA Regulation of Glutathione Homeostasis in Cancer Initiation, Progression and Therapy Resistance. MicroRNA.

[179] Lusini L., Tripodi S.A., Rossi R., Giannerini F., Giustarini D., del Vecchio M.T., Barbanti G., Cintorino M., Tosi P., Di Simplicio P. (2001). Altered glutathione anti-oxidant metabolism during tumor progression in human renal-cell carcinoma. Int. J. Cancer.

[180] Lau M.F., Chua K.H., Sabaratnam V., Kuppusamy U.R. (2020). Rosiglitazone enhances the apoptotic effect of 5-fluorouracil in colorectal cancer cells with high-glucose-induced glutathione. Sci. Prog..

[181] Bhowmick R., Sarkar R.R. (2020). Differential suitability of reactive oxygen species and the role of glutathione in regulating paradoxical behavior in gliomas: A mathematical perspective. PLoS ONE.

[182] Theodossiou T.A., Olsen C.E., Jonsson M., Kubin A., Hothersall J.S., Berg K. (2017). The diverse roles of glutathione-associated cell resistance against hypericin photodynamic therapy. Redox Biol..

[183] De Luca A., Parker L.J., Ang W.H., Rodolfo C., Gabbarini V., Hancock N.C., Palone F., Mazzetti A.P., Menin L., Morton C.J. (2019). A structure-based mechanism of cisplatin resistance mediated by glutathione transferase P1-1. Proc. Natl. Acad. Sci. USA.

[184] Alqarni M.H., Foudah A.I., Muharram M.M., Labrou N.E. (2021). The Interaction of Human Glutathione Transferase GSTA1-1 with Reactive Dyes. Molecules.

[185] Hamid A.A.A., Rahim R., Teo S.P. (2022). Pharmacovigilance and Its Importance for Primary Health Care Professionals. Korean J. Fam. Med..

[186] Song H., Pei X., Liu Z., Shen C., Sun J., Liu Y., Zhou L., Sun F., Xiao X. (2023). Pharmacovigilance in China: Evolution and future challenges. Br. J. Clin. Pharmacol..

[187] Hadi M.A., Neoh C.F., Zin R.M., Elrggal M.E., Cheema E. (2017). Pharmacovigilance: Pharmacists’ perspective on spontaneous adverse drug reaction reporting. Integr. Pharm. Res. Pract..

[188] Lorberbaum T., Nasir M., Keiser M.J., Vilar S., Hripcsak G., Tatonetti N.P. (2015). Systems pharmacology augments drug safety surveillance. Clin. Pharmacol. Ther..

[189] Jacob D., Marrón B., Ehrlich J., Rutherford P.A. (2013). Pharmacovigilance as a tool for safety and monitoring: A review of general issues and the specific challenges with end-stage renal failure patients. Drug Healthc. Patient Saf..

[190] Alzahrani T.F., Alotaibi S.M., Alzahrani A.A., Alzahrani A.F., Alturki L.E., Alshammari M.M., Alharbi R.A., Alanazi S.I., Alshammari W.Z., Algarni A.S. (2025). Exploring the Safety and Efficacy of Glutathione Supplementation for Skin Lightening: A Narrative Review. Cureus.

[191] Mackey T.K., Liang B.A. (2013). Improving global health governance to combat counterfeit medicines: A proposal for a UNODC-WHO-Interpol trilateral mechanism. BMC Med..

[192] Jackson G., Patel S., Khan S. (2012). Assessing the problem of counterfeit medications in the United Kingdom. Int. J. Clin. Pract..

[193] Pathak R., Gaur V., Sankrityayan H., Gogtay J. (2023). Tackling Counterfeit Drugs: The Challenges and Possibilities. Pharm. Med..

[194] Ozawa S., Evans D.R., Bessias S., Haynie D.G., Yemeke T.T., Laing S.K., Herrington J.E. (2018). Prevalence and Estimated Economic Burden of Substandard and Falsified Medicines in Low- and Middle-Income Countries: A Systematic Review and Meta-analysis. JAMA Netw. Open.

[195] Page M.J., McKenzie J.E., Bossuyt P.M., Boutron I., Hoffmann T.C., Mulrow C.D., Shamseer L., Tetzlaff J.M., Akl E.A., Brennan S.E. (2021). The PRISMA 2020 statement: An updated guideline for reporting systematic reviews. BMJ.

