# Supplementary material for: Glutathione in Skin Aging and Tissue Regeneration: A Systematic Review of Molecular Mechanisms, Redox Modulation, and Biomedical Implications
Source: Molecules. 2026 Mar 15;31(6):981. doi: 10.3390/molecules31060981 (PMC13029213; doi:10.3390/molecules31060981)
Supplement: Supplementary file 1 [file molecules-31-00981-s001.zip › molecules-4169331 - PRISMA_2020_checklist.pdf]

PRISMA 2020 Checklist

Section and Topic	Item #	Checklist item	Location where item is reported
**TITLE**			
Title	1	Glutathione in Skin Aging and Tissue Regeneration: Molecular Mechanisms, Redox Modulation, and Biomedical Implications — A Systematic Review	Page 1
**ABSTRACT**			
Abstract	2	Glutathione (GSH) is a central regulator of redox homeostasis, melanogenesis, and cellular repair, and has gained increasing attention in dermatology for its potential roles in skin brightening, anti-aging, and tissue regeneration. This systematic review aimed to evaluate the molecular, clinical, and translational evidence regarding the clinical applications of glutathione and safety across different delivery modalities. A comprehensive search of PubMed, Scopus, Web of Science, Embase, Cochrane Library, and Google Scholar was conducted for studies published between 2000 and 2025. We excluded reviews, meta-analyses, conference abstracts without full text, non-English articles, studies lacking primary data, and papers unrelated to dermatology or regenerative biology. A total of 194 studies meet the inclusion criteria, evaluating the effectiveness of glutathione in aesthetic dermatology and regenerative medicine. Risk of bias was assessed independently by two reviewers using validated tools appropriate for each study design. Randomized controlled trials were evaluated with the Cochrane RoB 2 tool across five domains (randomization, deviations from intended interventions, missing outcome data, outcome measurement, and selective reporting). Results were synthesized narratively due to the heterogeneity of study designs (in vitro, animal, and human studies), interventions, formulations, and outcome measures. Topical and oral glutathione demonstrated favorable effects on pigmentation, skin brightness, hydration, and oxidative stress markers. Injectable glutathione increased systemic levels rapidly, but was evaluated in fewer controlled studies. Glutathione S-transferases facilitate the conjugation of glutathione to electrophilic xenobiotics, thereby protecting proteins and nucleic acids from damage caused by electrophiles. Glutathione Peroxidase employs GSH as an electron donor to reduce hydrogen peroxide and lipid hydroperoxides, thus protecting membrane lipids, mitochondrial membranes, and DNA from oxidative damage. Glutathione facilitates the regeneration of other antioxidants, such as vitamin C and vitamin E, through redox cycling. A consistent correlation exists between reduced GSH levels and neuronal dysfunction. Elevated GSH levels enhance cellular resistance to oxidative stress and reduce apoptotic signaling. Despite the growing interest in glutathione's dermatological applications, current evidence is limited by short study durations, small sample sizes, and single-center designs. The significant variability in study design, formulations, dosages, and outcomes impedes the establishment of standardized therapeutic protocols. To elucidate the clinical significance of glutathione and enhance its dermatological uses, future research should focus on conducting well-designed randomized controlled trials, developing standardized formulations, and performing long-term safety assessments.<br>The review was not registered in a publicly accessible registry (e.g., PROSPERO).	Page 1
**INTRODUCTION**			
Rationale	3	The introduction explains that glutathione is a key regulator of redox homeostasis, melanogenesis, and cellular repair, and highlights the growing interest in its dermatological and regenerative applications. Existing evidence is fragmented across molecular, animal, and clinical studies, with substantial variability in formulations, delivery routes, and outcome measures. The review was conducted to synthesize this dispersed body of knowledge, clarify the therapeutic relevance and safety of glutathione, and address the lack of standardized clinical guidance.	Page 2-8
Objectives	4	The objective of the review was to evaluate the molecular mechanisms, clinical efficacy, delivery modalities, and safety of glutathione in skin aging and tissue regeneration. The review aimed to synthesize evidence from in vitro, animal, and human studies to determine how glutathione influences redox homeostasis, pigmentation, oxidative stress, and tissue repair, and to assess its translational relevance in dermatology and regenerative medicine.	Page 2
**METHODS**			
Eligibility criteria	5	Inclusion criteria comprised original in vitro, animal, and human studies evaluating glutathione or glutathione-related interventions in the context of skin aging, pigmentation, oxidative stress, or tissue regeneration. Eligible study designs included randomized controlled trials, non-randomized clinical studies, observational studies, mechanistic studies, and translational research published between 2000 and 2025. Exclusion criteria	Page 8

PRISMA 2020 Checklist

Section and Topic	Item #	Checklist item	Location where item is reported
		included review articles, meta-analyses, conference abstracts without full text, non-English publications, studies lacking primary data, and studies unrelated to dermatology or regenerative biology. For synthesis, studies were grouped according to delivery modality (topical, oral, injectable), mechanistic focus (redox regulation, antioxidant pathways, enzymatic activity), study type (in vitro, animal, human), and biomedical implications (cardiovascular homeostasis, respiratory defense and pulmonary redox balance, gastrointestinal integrity, neurodegeneration, and carcinogenesis) to allow structured narrative comparison. Studies were further categorized by advances in delivery systems (liposomes, nanoparticles, hydrogels) and by pharmacovigilance gaps.	
Information sources	6	The following electronic databases were searched: PubMed, Scopus, Web of Science, Embase, Cochrane Library, and Google Scholar. All databases were searched from January 2000 to December 2025. In addition, reference lists of relevant articles and reviews were manually screened to identify additional eligible studies. No study registries, organizational reports, or unpublished datasets were used as sources.	Page 8
Search strategy	7	A comprehensive literature search was performed across six electronic databases: PubMed/MEDLINE, Scopus, Web of Science Core Collection, Embase, Cochrane Library, and Google Scholar. The search covered studies published between January 2000 and September 2025. The search strategy combined Medical Subject Headings (MeSH) and free-text terms using Boolean operators (AND, OR). The core search string applied in PubMed was: "glutathione", "glutathione metabolism", "redox homeostasis", "skin rejuvenation", "skin aging", "tissue regeneration", "wound healing", "molecular mechanisms", "cellular detoxification", "bioavailability", "topical delivery", "clinical implications", "ethical standards" and "pharmacovigilance". Equivalent search strings were adapted for Scopus, Web of Science, Embase, and Cochrane Library according to each database's indexing system.	Page 8
Selection process	8	All records identified through database searches were screened in two stages: title/abstract screening followed by full-text assessment. Two reviewers independently screened each record and each full-text report to determine eligibility based on predefined inclusion and exclusion criteria. Discrepancies were resolved through discussion until consensus was reached. No automation tools, machine-learning classifiers, or AI-assisted screening systems were used in the selection process.	page 8
Data collection process	9	Data extraction was performed independently by two reviewers using a standardized data extraction form developed prior to the review process. All studies that met the eligibility criteria after full-text screening were included in the final synthesis (n = 194). Any discrepancies in extracted data were resolved through discussion until consensus was reached. For each included study, the following information was systematically collected:<br>• Study characteristics: author(s), year of publication, country, study design, sample size, and population type (human, animal, or in vitro)<br>• Intervention details: glutathione formulation, dosage, duration, and mode of administration (oral, topical, injectable, or experimental exposure)<br>• Outcomes measured: markers of skin aging and regeneration, including elasticity, hydration, collagen synthesis, oxidative stress parameters, photoprotection, and cellular repair indicators.<br>No authors were contacted for additional information, as all included studies provided sufficient methodological and outcome details for analysis.	pages 8-9
Data items	10a	Data were collected for all outcomes related to glutathione's molecular activity, antioxidant capacity, redox homeostasis, melanogenesis, tissue regeneration, and clinical skin parameters. Specifically, outcomes included biochemical markers (GSH/GSSG ratio, GPx, GST, ROS levels),	page 9

Section and Topic	Item #	Checklist item	Location where item is reported
		cellular responses (oxidative stress, mitochondrial function, collagen synthesis, inflammatory mediators), pigmentation outcomes (melanin content, tyrosinase activity), and clinical dermatological endpoints (skin brightness, hydration, elasticity, wrinkle depth, erythema, adverse effects). For each study, all results compatible with the predefined outcome domains were extracted, regardless of measurement method, time point, or analytical approach. When multiple measures or time points were reported, all relevant data were collected to ensure comprehensive synthesis. No selective outcome extraction criteria were applied.	
	10b	In addition to outcomes, data were collected on study characteristics including publication year, country, study design, model system (in vitro, animal, human), sample size, participant or cell-line characteristics, intervention details (glutathione formulation, dosage, delivery route, treatment duration), comparator type, and methodological features relevant to risk of bias. Information on funding sources and potential conflicts of interest was also extracted when reported. When data were missing, unclear, or inconsistently reported, assumptions were not made; instead, the information was recorded as 'not reported' or 'unclear'. No attempts were made to contact study authors for clarification.	page 9
Study risk of bias assessment	11	Risk of bias was assessed independently by two reviewers using validated tools appropriate for each study design. Randomized controlled trials were evaluated using the Cochrane Risk of Bias 2 (RoB 2) tool, which assesses bias arising from randomization, deviations from intended interventions, missing outcome data, outcome measurement, and selective reporting. Non-randomized clinical studies, animal studies, and in vitro studies were assessed using standardized methodological quality criteria tailored to each design. Each domain was rated as 'Low', 'Some Concerns', or 'High' risk of bias, and overall judgments were assigned accordingly. Disagreements between reviewers were resolved through discussion until consensus was reached. No automation tools or machine-assisted systems were used in the risk-of-bias assessment.	page 9
Effect measures	12	Because of substantial heterogeneity in study designs, outcome measures, and reporting formats, no single standardized effect measure was applied across studies. For clinical outcomes, results were presented using the effect measures reported by each study, including mean changes, percentage improvements, or qualitative assessments of skin parameters (e.g., pigmentation, hydration, elasticity, wrinkle depth). For biochemical and mechanistic outcomes, measures included changes in glutathione levels (GSH/GSSG ratio), antioxidant enzyme activity (GPx, GST), reactive oxygen species levels, melanin content, and molecular markers of oxidative stress or tissue repair. For in vitro and animal studies, results were summarized descriptively according to the metrics used by the original authors. No pooled effect estimates or comparative effect sizes (e.g., risk ratios, mean differences) were calculated because comparable quantitative data were unavailable.	page 9
Synthesis methods	13a	To determine which studies contributed to each synthesis, all included studies were tabulated according to key intervention characteristics, outcome domains, and biological systems. Studies were first categorized by model type (in vitro, animal, human) and by glutathione delivery modality (topical, oral, injectable). They were then mapped against the predefined thematic groups established in the eligibility criteria and synthesis plan, including: (1) tissue regeneration, (2) cardiovascular homeostasis, (3) respiratory defense and pulmonary redox balance, (4) gastrointestinal integrity, (5) neurodegeneration, (6) carcinogenesis, (7) dermatological and pharmacological implications, (8) advances in delivery systems (liposomes, nanoparticles, hydrogels), and (9) pharmacovigilance considerations. Only studies whose characteristics aligned with the conceptual and mechanistic focus of each synthesis domain were included in that section. This structured mapping ensured consistent allocation of studies and avoided overlap between synthesis groups.	Page 9
	13b	No statistical transformations or imputations were required because the review did not perform a quantitative synthesis. Data were extracted and presented in the format reported by the original studies. When studies reported outcomes using different units, scales, or measurement methods, the results were summarized descriptively without converting them to a common metric. Missing summary statistics (e.g., standard deviations, confidence intervals) were not estimated or inferred; such information was recorded as 'not reported'. When outcome data were presented graphically without numerical values, no attempts were made to digitize or approximate the results. All data were prepared for synthesis by grouping narratives according to study design, intervention characteristics, and thematic domains.	Not applicable

PRISMA 2020 Checklist

Section and Topic	Item #	Checklist item	Location where item is reported
	13c	Results from individual studies were tabulated using structured summary tables that captured key study characteristics, intervention details, outcome measures, and principal findings. Tables were organized by study type (in vitro, animal, human) and by thematic synthesis domains to facilitate comparison across biological systems and delivery modalities. Narrative summaries accompanied each table to contextualize findings. Visual displays, including conceptual diagrams and thematic flow figures, were used to illustrate mechanistic pathways, glutathione-related biological processes, and relationships between intervention types and observed outcomes. No statistical plots (e.g., forest plots) were generated because no meta-analysis was conducted.	Table 3
	13d	Given the substantial heterogeneity in study designs, model systems, glutathione formulations, delivery routes, and outcome measures, a quantitative synthesis (meta-analysis) was not feasible. Instead, a narrative synthesis approach was used. Studies were grouped thematically according to biological system, mechanistic pathway, and intervention characteristics, and findings were summarized descriptively within each synthesis domain. This approach allowed integration of molecular, preclinical, and clinical evidence while respecting differences in methodology and reporting. No statistical models, heterogeneity assessments, or meta-analytic software were used.	Page 9
	13e	Because the included studies varied substantially in design, model system, glutathione formulation, delivery route, and outcome measures, no formal statistical methods (such as subgroup analysis or meta-regression) were used to explore heterogeneity. Instead, potential sources of heterogeneity were examined qualitatively by comparing findings across predefined thematic domains (e.g., tissue regeneration, cardiovascular homeostasis, respiratory defense, gastrointestinal integrity, neurodegeneration, carcinogenesis), delivery modalities (topical, oral, injectable), and study types (in vitro, animal, human). Differences in methodological quality, dosage, and treatment duration were also considered narratively when interpreting variability in results.	Page 9
	13f	No sensitivity analyses were conducted because no quantitative synthesis or pooled effect estimates were generated. The robustness of conclusions was assessed qualitatively by examining the consistency of findings across independent studies, biological models, and mechanistic pathways.	Not applicable
Reporting bias assessment	14	The potential risk of bias due to missing results was assessed qualitatively by examining the completeness of outcome reporting in each study and by comparing reported outcomes with the study objectives and methods. Selective reporting was considered likely when prespecified outcomes were mentioned in the methods but not fully presented in the results, or when incomplete or inconsistent reporting patterns were observed. No automation tools were used to assess reporting bias.	Table 4
Certainty assessment	15	Because the review synthesized heterogeneous evidence from in vitro, animal, and human studies and did not perform a quantitative meta-analysis, formal frameworks for rating certainty of evidence (such as GRADE) were not applied. Instead, certainty in the body of evidence for each outcome was assessed qualitatively by considering the consistency of findings across independent studies, the methodological quality and risk-of-bias assessments, the biological plausibility of mechanistic pathways, and the coherence between preclinical and clinical results. No automation tools were used to assess certainty.	Not applicable
**RESULTS**			
Study selection	16a	The database search yielded 2,538 records: PubMed (n = 1,33), Embase/Scopus (n = 348), Web of Science (n = 315), Cochrane Library (n = 273), and Google Scholar (n = 216). After removing 214 duplicates, 2,324 records remained for screening. Of these, 669 were automatically marked as ineligible based on database filters, and 43 were excluded for other reasons (e.g., non-retrievable records, incomplete metadata). A	pages 9-10

Section and Topic	Item #	Checklist item	Location where item is reported
		total of 1,619 records proceeded to title and abstract screening.<br>During the screening phase, 1147 records were excluded for failing to meet the predefined eligibility criteria. The remaining 472 full-text articles were assessed for eligibility. Following a detailed evaluation, 194 studies met all inclusion criteria and were included in the final synthesis. Reasons for full-text exclusion were documented and are summarized in the PRISMA flow diagram (Figure 5).	
	16b	A total of 472 full-text articles were assessed for eligibility. Of these, 278 studies were excluded after detailed evaluation. Several articles appeared initially eligible based on title and abstract, but were excluded at the full-text stage for the following reasons:<br>• Not meeting the population, intervention, or outcome criteria (e.g., studies evaluating glutathione in systemic metabolic or hepatic conditions without relevance to dermatology, redox biology, or tissue regeneration).<br>• Absence of glutathione or glutathione-related interventions, despite initial indexing suggesting otherwise.<br>• Study type not eligible, including narrative reviews, commentaries, conference abstracts without primary data, or methodological papers.<br>• Insufficient or non-extractable data, such as incomplete reporting of outcomes, lack of quantitative or qualitative results, or missing methodological details.<br>• Irrelevant focus, including studies centered on unrelated antioxidants, metabolic pathways, or pharmacological agents.<br>• Full text not available in English, as required by the predefined eligibility criteria.<br>• Duplicate publications or secondary analyses of previously included datasets.	page 10
Study characteristics	17	Each included study was cited and its key characteristics were extracted and summarized in Table 3	Table 3
Risk of bias in studies	18	Risk-of-bias assessments were conducted for all 194 included studies using tools appropriate to each study design. For randomized clinical trials, risk of bias was evaluated with the Cochrane RoB 2 tool across its standard domains. Non-randomized clinical studies, animal studies, and in vitro studies were appraised using predefined methodological quality criteria tailored to each design, focusing on selection methods, intervention reporting, outcome measurement, and data completeness. For each study, domain-level judgments and an overall risk-of-bias rating (Low, Some Concerns, or High) were assigned. The detailed risk-of-bias assessments for individual studies are presented in the Supplementary Table.	Table 4
Results of individual studies	19	Because of substantial heterogeneity in study designs, model systems, glutathione formulations, delivery routes, and outcome measures, uniform summary statistics and standardized effect estimates could not be generated across studies.	Not applicable
Results of syntheses	20a	For each synthesis domain, we summarized the characteristics of contributing studies and their risk-of-bias profiles. The syntheses included heterogeneous evidence from in vitro experiments, animal models, and human clinical studies, with substantial variation in glutathione formulations, delivery routes, dosages, and outcome measures. In tissue regeneration studies, most work has been preclinical (in vitro and animal), typically using reduced glutathione or glutathione-enhancing compounds. These studies generally demonstrated consistent mechanistic effects but frequently exhibited methodological limitations such as small sample sizes, limited blinding, or incomplete reporting, resulting in overall ratings of ‘Some Concerns’. In syntheses on cardiovascular, respiratory, gastrointestinal, and neurodegenerative diseases, studies varied widely in model systems and outcome metrics. Animal studies often showed coherent antioxidant and cytoprotective effects but were commonly rated as ‘Some Concerns’ due to unclear allocation methods and selective reporting. Human studies in these domains were fewer and generally small, with mixed risk-of-bias profiles. In the carcinogenesis synthesis, included studies were predominantly mechanistic and in vitro, with high variability in experimental conditions. Although mechanistic consistency was observed, many studies lacked detailed methodological reporting,	Table 4

PRISMA 2020 Checklist

Section and Topic	Item #	Checklist item	Location where item is reported
		raising concerns about the risk of bias. In the dermatological and pharmacological applications, human clinical studies were more prevalent. Randomized controlled trials generally demonstrated lower risk of bias, while non-randomized studies showed greater variability in methodological rigor. Across all syntheses, risk-of-bias assessments reflected the diversity of study designs: randomized clinical trials tended to have lower risk, whereas in vitro and animal studies frequently lacked methodological detail. These patterns were considered when interpreting the strength and consistency of evidence within each synthesis domain.	
	20b	No statistical syntheses or meta-analyses were conducted due to substantial heterogeneity in study designs, model systems, glutathione formulations, delivery routes, and outcome measures. As a result, no pooled summary estimates, confidence intervals, or measures of statistical heterogeneity (e.g., I^2) were generated. For each synthesis domain, results were summarized narratively, highlighting the direction and consistency of effects across studies. When individual studies compared intervention and control groups, the direction of effect (e.g., improvement, no change, or mixed findings) was described qualitatively based on the outcomes reported by the original authors. No quantitative effect estimates were calculated or combined.	Not applicable
	20c	No formal statistical investigations of heterogeneity (such as subgroup analyses, sensitivity analyses, or meta-regression) were conducted because no quantitative syntheses or pooled effect estimates were generated. However, potential sources of heterogeneity were explored qualitatively within each synthesis domain. Across syntheses, heterogeneity was examined by comparing results according to study design (in vitro, animal, human), glutathione formulation and delivery route (topical, oral, injectable), dosage, treatment duration, and outcome measurement methods. Differences in methodological quality and risk-of-bias profiles were also considered when interpreting variability in findings. Narrative comparisons highlighted that variations in experimental conditions, biological models, and reporting completeness contributed substantially to differences in observed effects. These qualitative assessments informed the interpretation of consistency and strength of evidence within each synthesis domain.	Not applicable
	20d	No sensitivity analyses were conducted because no quantitative syntheses or pooled effect estimates were generated. The substantial heterogeneity in study designs, model systems, glutathione formulations, delivery routes, and outcome measures precluded conducting statistical sensitivity analyses. Instead, robustness of the narrative syntheses was evaluated qualitatively by examining the consistency of findings across independent studies, biological models, and methodological quality levels. Patterns that remained stable across multiple study types or across studies with a lower risk of bias were considered more reliable.	Not applicable
Reporting biases	21	No formal statistical assessments of reporting bias (such as funnel plot asymmetry or regression-based tests) were conducted because no quantitative syntheses or pooled effect estimates were generated. However, the potential risk of bias due to missing results was examined qualitatively within each synthesis domain. Across syntheses, selective reporting was assessed by comparing the outcomes prespecified in each study's methods with those reported in the results. In vitro and animal studies frequently lacked complete numerical reporting or omitted secondary outcomes, contributing to concerns about reporting bias. Human clinical studies generally provided more structured reporting, although incomplete disclosure of adverse events or secondary endpoints was noted in some cases. In the tissue regeneration and mechanistic syntheses, reporting bias was more likely due to the predominance of preclinical studies with variable reporting standards. In the dermatology and clinical application syntheses, the risk of reporting bias was lower but still present when studies selectively emphasized positive findings or provided limited methodological detail. These qualitative assessments were incorporated into the interpretation of each synthesis, with greater caution applied in domains where incomplete or selective reporting was more prevalent.	Not applicable
Certainty of evidence	22	No formal certainty-of-evidence framework (such as GRADE) was applied because the review synthesized heterogeneous evidence from in vitro, animal, and human studies and did not include quantitative effect estimates. Instead, certainty in the body of evidence for each outcome was assessed qualitatively. Across outcomes, higher confidence was assigned when findings were consistent across multiple independent studies, supported by coherent mechanistic pathways, and derived from studies with a lower risk of bias. Outcomes supported primarily by in vitro or small animal studies, or by studies with incomplete reporting, were judged to have lower certainty. For dermatological and clinical	Not applicable

Section and Topic	Item #	Checklist item	Location where item is reported
		outcomes, certainty was generally moderate, reflecting the presence of randomized or controlled human studies, although sample sizes were often small. For mechanistic, regenerative, and systemic outcomes, certainty was lower due to reliance on preclinical models and variable methodological rigor. These qualitative certainty assessments were integrated into the interpretation of each synthesis and are summarized narratively within the Results section.	
**DISCUSSION**			
Discussion	23a	The findings of this review align with and expand upon existing evidence regarding the biological and clinical roles of glutathione. Across in vitro, animal, and human studies, the results consistently support glutathione's central function as a master antioxidant, redox regulator, and cytoprotective molecule. These observations are consistent with prior mechanistic literature describing glutathione as a key determinant of cellular resilience, mitochondrial stability, and detoxification capacity. In dermatology, the included clinical studies reinforce earlier reports that glutathione contributes to skin brightening, modulates melanogenesis, and attenuates oxidative stress–related aging processes. These findings are consistent with previous clinical and cosmetic research, although the magnitude of benefit varies across formulations and delivery routes. In tissue regeneration and systemic domains (cardiovascular, respiratory, gastrointestinal, and neurodegenerative), the results corroborate established mechanistic pathways demonstrating glutathione's involvement in maintaining cellular homeostasis, reducing inflammatory signaling, and protecting against oxidative injury. However, much of this evidence remains preclinical, and its translation into clinical outcomes remains limited. In carcinogenesis, the review's findings reflect the dual role of glutathione described in prior literature: protective in normal cells but potentially supportive of survival pathways in malignant cells. This complexity mirrors existing debates in oncology research and underscores the need for context-specific interpretation. Overall, the results of this review are broadly consistent with the wider body of evidence, strengthening the understanding that glutathione plays a multifaceted role across biological systems. At the same time, the heterogeneity of study designs and the predominance of preclinical data in several domains highlight the need for more standardized, well-designed clinical studies to clarify optimal formulations, dosing strategies, and therapeutic applications.	Pages 10-20
	23b	The evidence included in this review presents several important limitations that affect the strength and generalizability of the conclusions. First, a substantial proportion of the studies were preclinical (in vitro or animal), which, although valuable for mechanistic insight, limit the direct applicability of findings to human physiology and clinical outcomes. Many of these studies used heterogeneous experimental conditions, variable glutathione formulations, and inconsistent outcome measures, making comparisons across studies challenging. Second, the number of well-designed human clinical studies was relatively small, and sample sizes were often limited. Several clinical studies lacked rigorous methodological features, such as randomization, blinding, or adequate control groups, raising concerns about internal validity. Reporting of adverse events and secondary outcomes was inconsistent, reducing the ability to fully assess safety and broader clinical effects. Third, across all study types, reporting quality varied considerably. Many preclinical studies did not provide complete numerical data, detailed methodological descriptions, or standardized outcome metrics. This incomplete reporting increased the risk of selective outcome reporting and limited the ability to extract comparable summary statistics. Fourth, heterogeneity in glutathione formulations (reduced glutathione, precursors, conjugates, delivery systems), dosing regimens, and treatment durations further limited the ability to synthesize quantitative results or draw formulation-specific conclusions. Finally, publication bias cannot be excluded, particularly in domains where positive mechanistic findings are more likely to be published than neutral or negative results. The predominance of small, single-center studies also raises concerns about reproducibility. Taken together, these limitations highlight the need for more standardized, methodologically robust, and transparently reported studies to clarify the therapeutic potential, optimal formulations, and safety profile of glutathione across biological systems.	Table 1, 2
	23c	Several limitations of the review processes should be acknowledged. First, although the search strategy was comprehensive and covered multiple major databases, the review was restricted to studies published in English. This language restriction may have led to the exclusion of relevant evidence published in other languages. Additionally, while Google Scholar was included to broaden coverage, its indexing heterogeneity and limited filtering capabilities may have resulted in missed or non-retrievable records. Second, screening and data extraction were performed using a structured, predefined approach, but the large volume of records increased the likelihood of human error during title/abstract screening and full-text assessment. Although efforts were made to ensure consistency, dual independent screening was not feasible at all stages due to the dataset's scale. Third, the extraction of numerical data was limited by the reporting quality of the included studies. Many preclinical studies did not provide extractable summary statistics, and no attempts were made to digitize figures or impute missing values. As a result, the ability to	Page 24

PRISMA 2020 Checklist

Section and Topic	Item #	Checklist item	Location where item is reported
		compare outcomes across studies was constrained by the variability and incompleteness of reported data. Finally, because no meta-analysis was conducted, the synthesis relied on narrative integration of heterogeneous evidence. While appropriate for the scope and diversity of the included studies, narrative synthesis is inherently more susceptible to interpretive bias than quantitative approaches. These limitations highlight the need for more standardized reporting, validated assessment tools for preclinical research, and greater methodological transparency to strengthen future evidence syntheses in this field.	
	23d	The findings of this review suggest several implications for practice, policy, and future research. From a practical perspective, the evidence supports the biological relevance of glutathione as a central regulator of redox balance, cellular protection, and tissue integrity. In dermatology, the available clinical studies indicate potential benefits for skin brightening, oxidative stress reduction, and anti-aging applications, although variability in formulations and dosing limits the ability to recommend standardized therapeutic protocols. Clinicians and practitioners should therefore interpret existing evidence cautiously and consider glutathione as a complementary intervention rather than a fully established therapeutic option. From a policy standpoint, the heterogeneity of formulations, delivery systems, and regulatory classifications underscores the need for clearer guidelines on quality control, labeling, and safety monitoring for glutathione-based products. Standardizing manufacturing practices and transparently reporting composition, bioavailability, and stability would support more consistent clinical use and facilitate regulatory oversight. For future research, the review underscores the need for well-designed, adequately powered clinical trials that use standardized glutathione formulations, clearly defined dosing regimens, and validated outcome measures. Greater methodological rigor is particularly important in domains where evidence is predominantly preclinical, such as tissue regeneration, systemic antioxidant effects, and mechanistic pathways. Studies should also prioritize comprehensive reporting of adverse events, pharmacokinetics, and long-term safety. Additionally, translational research that bridges mechanistic findings to clinical endpoints would help clarify the therapeutic potential of glutathione across biological systems.	Page 25
**OTHER INFORMATION**			
Registration and protocol	24a	This review was not registered in a prospective review registry such as PROSPERO or OSF Registries. At the time the review was initiated, no formal protocol was publicly recorded.	Not applicable
	24b	No review protocol was prepared for this work, and no protocol is publicly available. The review was conducted without prior registration or publication of a predefined methodological protocol.	Not applicable
	24c	Because the review was not registered and no formal protocol was prepared, there were no amendments to previously documented methods or procedures. All methodological decisions, including eligibility criteria, search strategy, data extraction approach, and synthesis methods, were developed and applied throughout the review.	Not applicable
Support	25	This review did not receive any specific financial or non-financial support from funding agencies, institutions, or commercial entities. No external sponsors were involved in the conception, design, data collection, analysis, interpretation, or writing of the review. All work was conducted independently by the authors.	Page 26
Competing interests	26	The authors declare no competing interests. No financial, personal, academic, or institutional relationships influenced the conception, conduct, analysis, or reporting of this review.	Page 26
Availability of data, code and other materials	27	No materials from this review are publicly available. Template data-collection forms, extracted data, analytic datasets, and any other materials used during the review were not deposited in a public repository. All data were derived directly from the published studies included in the review, and no additional datasets or analytic code were generated.	Page 26

From: Page MJ, McKenzie JE, Bossuyt PM, Boutron I, Hoffmann TC, Mulrow CD, et al. The PRISMA 2020 statement: an updated guideline for reporting systematic reviews. BMJ 2021;372:n71. doi: 10.1136/bmj.n71. This work is licensed under CC BY 4.0. To view a copy of this license, visit https://creativecommons.org/licenses/by/4.0/
