# Supplementary material for: Glutathione in Skin Aging and Tissue Regeneration: A Systematic Review of Molecular Mechanisms, Redox Modulation, and Biomedical Implications
Source: Molecules. 2026 Mar 15;31(6):981. doi: 10.3390/molecules31060981 (PMC13029213; doi:10.3390/molecules31060981)
Supplement: Supplementary file 1 [file molecules-31-00981-s001.zip › molecules-4169331 - Table S1 Characteristics of Included Studies.pdf]

Table S1. Characteristics of Included Studies (n=194)

No	Study ID	Study design	Population / model	Intervention / exposure	Comparator	Outcomes
1.	Ansary et al., 2021	Narrative review	N/A	UV-induced inflammatory pathways	N/A	Mechanisms of photoaging
2.	Guerrero-Navarro et al., 2024	In vitro experimental study	Human dermal fibroblasts	UV + particulate matter exposure	Untreated cells	Autophagy markers, senescence
3.	Soheilifar et al., 2022	Narrative review	N/A	Non-coding RNAs in photoaging	N/A	Mechanistic pathways
4.	Fisher et al., 2002	Narrative mechanistic review	N/A	UV and intrinsic aging pathways	N/A	Collagen degradation, MMPs
5.	Parrado et al., 2019	Narrative review	N/A	Environmental stressors	N/A	Oxidative stress, aging
6.	Bouayed & Bohn, 2010	Narrative review	N/A	Exogenous antioxidants	N/A	Redox balance
7.	Stanescu et al., 2025	Systematic review	N/A	Carotenoids	N/A	Skin aging pathway
8.	Kim et al., 2023	In vitro experimental	Stem cells	Glutathione regeneration assays	Baseline cells	GSH regeneration capacity
9.	Labunskyy & Gladyshev, 2013	Narrative mechanistic review	N/A	ROS signaling	N/A	Aging pathway
10.	Süntar et al., 2021	Narrative review	N/A	Nrf2 pathway	N/A	Wound healing mechanisms
11.	Kalinina et al., 2014	Narrative mechanistic review	N/A	GSH, GST, glutaredoxin	N/A	Redox regulation
12.	Hunt et al., 2024	Narrative review	N/A	ROS in wound healing	N/A	Cellular roles of ROS
13.	Aquilano et al., 2014	Narrative review	N/A	Glutathione in redox signaling	N/A	Mechanistic pathway
14.	Gasmi et al., 2024	Narrative review	N/A	GSH biosynthesis & metabolism	N/A	Physiological functions
15.	Berndt & Lillig, 2017	Narrative mechanistic review	N/A	GSH, glutaredoxins, iron	N/A	Redox regulation
16.	Riskowski et al., 2019	In vitro biochemical study	Enzyme systems	GSH-dependent quantum dot synthesis	No enzyme / no GSH	Fluorescence, catalytic activity
17.	Scirè et al., 2019	Narrative mechanistic review	N/A	GSH compartmentalization	N/A	Redox regulation

18.	Cacciatore et al., 2010	Experimental (in vitro + chemical synthesis)	Cell lines	GSH-enhancing prodrugs	No prodrug	Intracellular GSH levels
19.	Vázquez-Meza et al., 2023	Narrative review	N/A	GSH transport	N/A	Pathophysiolo gy
20.	Diaz-Vivancos et al., 2015	Narrative mechanistic review	N/A	GSH & oxidative stress	N/A	Cell proliferation pathways
21.	Chakraborty et al., 2022	Narrative mechanistic review	N/A	S-denitrosylation, GSH–redoxin crosstalk	N/A	Redox signaling pathways
22.	Bettendorff, 2022	Narrative biochemical review	N/A	Reduced nucleotides, thiols, O_2	N/A	Redox balance
23.	Maestri et al., 2021	Experimental + transcriptomi c analysis	Human intestinal mucosa	Short-term caloric restriction	Baseline mucosa	Telomere length, immunity markers
24.	Narayanankutt y et al., 2019	Narrative review	N/A	GSH in carcinogenesis	N/A	Dual roles of GSH
25.	Dergousova et al., 2017	In vitro biochemical study	Enzyme assays (Na,K-ATP ase)	Redox modification of cysteine residues	Untreate d enzyme	ATPase activity
26.	Mazari et al., 2023	Narrative review	N/A	GSTs in health and disease	N/A	Mechanistic roles
27.	Anashkina et al., 2023	In vitro + comparative biochemical study	Hemoglobi n from multiple mammals	GSH binding / glutathionylation	Species compari son	Binding affinity, structural conservation
28.	Li et al., 2012	Narrative review	N/A	GSH in endothelial biology	N/A	Brain microvascular homeostasis
29.	Kim et al., 2024	In vitro biochemical study	GSTO1 enzyme assays	Redox-sensitive cysteine regulation	Mutant or untreate d enzyme	Enzyme activity, redox response
30.	Lubos et al., 2011	Narrative mechanistic review	N/A	GPx-1 biology	N/A	Redox mechanisms
31.	Chang et al., 2020	Narrative review	N/A	GPx3 in cancer	N/A	Tumor biology
32.	Handy et al., 2009	In vitro + mechanistic study	Cell lines	GPx-1 modulation	Baseline cells	Mitochondrial function, ROS
33.	Sands et al., 2018	In vitro chemical synthesis +	Chemical compounds	GPx-mimetic seleninate esters	No compou nd	Antioxidant activity

		biological assays	+ cell assays			
34.	Vašková et al., 2023	Narrative review	N/A	GSH-related enzymes	N/A	Redox pathway
35.	Lushchak, 2012	Narrative review	N/A	GSH homeostasis	N/A	Medical implications
36.	Aoyama & Nakaki, 2015	Narrative mechanistic review	N/A	EAAC1 and GSH	N/A	Redox homeostasis
37.	Chai & Mieyal, 2023	Narrative review	N/A	GSH & glutaredoxin	N/A	Redox signaling
38.	Chełchowska et al., 2025	Observational clinical pilot study	Children with ASD	Measurement of GSH, Nrf2	Healthy controls	Oxidative stress markers
39.	Dong et al., 2018	Narrative review	N/A	GST-π in cancer therapy	N/A	Mechanistic roles
40.	Dringen et al., 2015	Narrative mechanistic review	N/A	GSH detoxification in astrocytes	N/A	Redox metabolism
41.	Hong et al., 2006	In vitro experimental	Rat brain microvessel endothelial cells	GSH depletion (BSO)	Untreated cells	P-gp expression, oxidative stress
42.	Presnell et al., 2013	Computational modeling study	In silico	GSH redox simulations	N/A	Oxidative stress modeling
43.	Hushcha et al., 2021	Narrative review	N/A	microRNAs in melanogenesis	N/A	Regulatory pathway
44.	Kondo & Hearing, 2011	Narrative review	N/A	Melanocyte regulation	N/A	Pigmentation mechanisms
45.	Putri et al., 2025	In silico + peptide design	Computational peptide modeling	Anti-melanogenesis peptide optimization	N/A	Binding affinity, predicted activity
46.	Dilokthornsakul et al., 2019	Systematic review	N/A	Glutathione for skin color	N/A	Clinical effects
47.	Pillaiyar et al., 2018	Narrative review	N/A	Melanogenesis inhibitors	N/A	Mechanisms, compounds
48.	Qian et al., 2020	Narrative review	N/A	Natural skin-whitening compounds	N/A	Melanogenesis pathway
49.	Schmidt et al., 2019	In vivo + in vitro experimental	Mouse wound model + keratinocytes	Cold plasma exposure	Untreated wounds/ cells	Nrf2 activation, inflammation, healing rate
50.	Leavitt et al., 2016	Narrative review	N/A	Scarless healing mechanisms	N/A	Cell signaling, regeneration
51.	Stanescu et al., 2025	Systematic review	N/A	Micronutrition & nutraceuticals	N/A	Wound healing outcomes

52.	Lekas et al., 2006	Narrative review	N/A	Growth factor-induced neovascularization	N/A	Therapeutic angiogenesis
53.	Ahn et al., 2008	Narrative review	N/A	Therapeutic angiogenesis	N/A	Ischemic heart disease
54.	Pessoa et al., 2016	In vivo experimental	Diabetic mice	Oral antioxidants (mixture)	Diabetic untreated mice	Wound closure, inflammation
55.	Bains & Bains, 2015	Narrative review	N/A	GSH & periodontal health	N/A	Redox balance
56.	Arnal-Forné & Borrás, 2025	Systematic review + meta-analysis	N/A	Redox-targeting therapies	N/A	Wound healing outcomes
57.	Lin & Lai, 2024	Narrative review	N/A	Scarring mechanisms	N/A	Fibrosis, therapies
58.	Sangha et al., 2024	Narrative review	N/A	Wound healing & scarring	N/A	Management strategies
59.	Stanescu et al., 2025	Narrative review	N/A	Emotional impact of scars	N/A	Patient education
60.	Giustarini et al., 2023	Narrative review	N/A	Strategies to increase GSH	N/A	Mechanistic pathways
61.	Świderska-Kołacz et al., 2021	In vivo experimental	Mice (liver & kidney)	Algae supplementation	Standard diet	GSH levels, GSH enzyme activity
62.	Al-Temimi et al., 2023	Narrative review	N/A	GSH extraction & quantification	N/A	Methods overview
63.	Minich & Brown, 2019	Narrative review	N/A	Dietary nutrients affecting GSH	N/A	Mechanistic & nutritional roles
64.	Liebman & Le, 2021	Narrative review	N/A	Sulforaphane, NRF2	N/A	Oxidative stress pathways
65.	Nogales et al., 2013	In vivo experimental	Rat offspring	Selenium supplementation	Standard diet	Growth, oxidative balance, GPx
66.	Dogan et al., 2016	In vitro biochemical + food analysis	Edible mushrooms	Measurement of GSH, Se, MDA	Different species	Antioxidant content
67.	Baldelli et al., 2019	Narrative review	N/A	GSH & nitric oxide in muscle	N/A	Muscle use/disuse pathways
68.	Zhou et al., 2002	In vivo experimental	Hypertensive rats	Inhibition of GSH synthase	Healthy rats	NOS expression, BP
69.	Chen et al., 2021	In vitro chemical probe study	Cell lines	NO–GSH fluorescent probe	No probe	Imaging of signaling pathways

70.	Niki, 2011	Narrative review	N/A	Free radicals & LDL oxidation	N/A	Atherosclerosis mechanisms
71.	Victor et al., 2009	Narrative review	N/A	Oxidative stress & endothelial dysfunction	N/A	Atherosclerosis pathways
72.	Roşian et al., 2025	Narrative review	N/A	Bioactive compounds in atherosclerosis	N/A	Antioxidant & anti-inflammatory roles
73.	He et al., 2020	In vivo + in vitro experimental	VSMCs + mice	Choline activation of M3AChR & Nrf2	Untreated controls	Phenotypic switching, remodeling
74.	Matsumori, 2023	Narrative review	N/A	Mitochondrial permeability transition pore	N/A	Ischemia-reperfusion injury
75.	Baines, 2009	Narrative mechanistic review	N/A	Mitochondrial permeability transition pore	N/A	Ischemia-reperfusion injury
76.	Kahl et al., 2018	In vivo experimental	Mouse brain ischemia mode	Complex I inhibition + GSH modulation	Sham controls	Bioenergetic failure, ROS
77.	Hu et al., 2019	Narrative review	N/A	Mitochondrial DNA in I/R injury	N/A	Mechanistic pathways
78.	Jassem et al., 2002	Narrative review	N/A	Mitochondria in I/R injury	N/A	Transplantation mechanisms
79.	Rashdan et al., 2020	Narrative review	N/A	S-glutathionylation	N/A	Cardiovascular redox regulation
80.	Rozanski & Xu, 2002	In vivo experimental	Post-MI rat hearts	GSH modulation	Healthy controls	K^+ channel remodeling
81.	Myszor & Gudmundsson, 2023	Narrative review	N/A	Innate immunity modulation	N/A	Host-directed therapy pathway
82.	Vareille et al., 2011	Narrative review	N/A	Airway epithelium & viral defense	N/A	Immune mechanisms
83.	Chen et al., 2024	Narrative mechanistic review	N/A	Mitochondrial glutathione	N/A	Redox homeostasis
84.	Cipollina et al., 2022	In vitro experimental	Bronchial epithelial cells	Cigarette smoke extract	Untreated cells	Oxidative stress markers
85.	Koike et al., 2007	In vivo experimental	Mice	GSH redox modulation	Wild-type controls	Airway inflammation, AHR
86.	Pekovic-Vaughan et al., 2014	In vivo + in vitro experimental	Mice + lung cells	Circadian NRF2/GSH pathway activation	Clock-disrupted or untreated	Fibrosis markers, antioxidant response

87.	Zuo & Wijegunawardana, 2021	Narrative review	N/A	ROS & inflammation in lung disease	N/A	Redox mechanisms
88.	Reddy, 2008	Narrative review	N/A	ARE/Nrf2 in airway disease	N/A	Oxidative stress pathways
89.	van de Wetering et al., 2021	Narrative review	N/A	GSTs in asthma & COPD	N/A	Genetic susceptibility
90.	Tasaka et al., 2008	Narrative review	N/A	Oxidants in ARDS	N/A	Redox signaling
91.	Di Tommaso et al., 2021	Narrative review	N/A	Intestinal barrier	N/A	Health & disease mechanisms
92.	Young, 2012	Narrative review	N/A	Gut microbiota	N/A	Health & disease
93.	Sulaiman et al., 2024	Narrative review	N/A	Oral/gut dysbiosis & cancer	N/A	Microbiota pathways
94.	Yu et al., 2024	Narrative review	N/A	Gut phageome	N/A	Microbiota interactions
95.	Xiang et al., 2022	In vivo experimental	Weaned piglets	Oral glutathione	No GSH	Intestinal barrier, antioxidant capacity
96.	Shang et al., 2016	In vivo experimental	Rats with AKI	Downregulation of GSH biosynthesis	Healthy controls	Oxidative stress, liver dysfunction
97.	Bonetti et al., 2024	In vivo + in vitro experimental	Th17 cells + mouse models	GSH/mitochondrial IL-22 axis	Knockout or untreated	Intestinal inflammation, mitochondrial function
98.	Wolozin & Behl, 2000	Narrative review	N/A	Protein aggregates in neurodegeneration	N/A	Mechanistic pathways
99.	Ren et al., 2017	Narrative mechanistic review	N/A	Thioredoxin & GSH systems	N/A	CNS redox signaling
100.	Sabens Liedhegner et al., 2012	Narrative mechanistic review	N/A	S-glutathionylation in neurodegeneration	N/A	Redox dysregulation
101.	Lana et al., 2024	Narrative review	N/A	Nebulized glutathione	N/A	Antioxidant effects in neurodegeneration
102.	Carvalho et al., 2014	Narrative review	N/A	GSH in multiple sclerosis	N/A	Redox & immune mechanisms

103.	Liu et al., 2004	Narrative review	N/A	GSH metabolism in aging & Alzheimer's	N/A	Redox dysregulation
104.	Chełchowska et al., 2025	Observational clinical pilot	Children with ASD	Measurement of GSH & Nrf2	Healthy controls	Oxidative stress markers
105.	Garcia-Bonilla et al., 2014	Narrative review	N/A	Immune mechanisms in ischemic tolerance	N/A	Neuroinflammation pathways
106.	Aoyama & Nakaki, 2013	Narrative review	N/A	Impaired GSH synthesis	N/A	Neurodegeneration mechanisms
107.	Valenti et al., 2023	Narrative review	N/A	GSH in cancer progression	N/A	Chemoresistance pathways
108.	Kennedy et al., 2020	Narrative review	N/A	GSH in cancer	N/A	Mechanisms & therapies
109.	Tamas et al., 2021	Observational clinical study	Patients with facial skin tumors	Clinical vs histological diagnosis	N/A	Diagnostic concordance
110.	Traverso et al., 2013	Narrative review	N/A	GSH in cancer progression	N/A	Redox & chemoresistance
111.	Bansal & Simon, 2018	Narrative mechanistic review	N/A	GSH metabolism in cancer	N/A	Treatment resistance
112.	Sekhar et al., 2022	In vitro + in vivo experimental	Thyroid cancer cells + mouse xenografts	GPx4 inhibition	Untreated controls	Ferroptosis, mTOR suppression
113.	Gronau et al., 2003	Observational clinical	Oral SCC patients	GSTM1 enzyme levels	Healthy tissue	Enzyme activity, genotype correlation
114.	Hu et al., 2024	Meta-analysis	N/A	GST gene variants	N/A	Cancer risk
115.	Li et al., 2021	Narrative review	N/A	GSH-responsive nanodrugs	N/A	Drug design principles
116.	Hash et al., 2025	Narrative review	N/A	AI in skincare	N/A	Personalized regimens
117.	Frasier et al., 2024	Narrative review	N/A	Wearable skin monitoring	N/A	Real-time dermatology
118.	Singh et al., 2024	Narrative review	N/A	AI in clinical pharmacology	N/A	Benefits & challenges
119.	Elder et al., 2021	Narrative review	N/A	AI in cosmetic dermatology	N/A	Current & future trends
120.	Nahm et al., 2025	Narrative review	N/A	Exosomes in dermatology	N/A	Clinical applications
121.	Torres et al., 2024	Narrative review	N/A	Topical vehicles/bases	N/A	Formulation characteristics

122.	Mayba & Gooderham, 2018	Narrative review	N/A	Topical vehicle formulations	N/A	Clinical relevance
123.	Buonocore et al., 2016	Non-randomized clinical bioavailability study	Healthy adults	Orobuccal glutathione	None	Plasma GSH levels
124.	Watanabe et al., 2014	Randomized, double-blind, placebo-controlled clinical trial	Healthy women	Topical oxidized glutathione	Placebo cream	Skin whitening, hydration
125.	Grandi et al., 2019	Randomized, double-blind, placebo-controlled clinical trial	Adults	S-acyl glutathione 2% cream	Placebo	UVB-induced erythema
126.	Cui et al., 2024	Non-randomized in vivo + in vitro	Volunteers + cell models	Topical GSH precursors	None	Oxidative stress, TEWL
127.	Arjinpathana & Asawanonda, 2012	Randomized, double-blind, placebo-controlled clinical trial	Adults	Oral glutathione	Placebo	Melanin index
128.	Handog et al., 2016	Open-label, single-arm clinical trial	Filipino women	Oral glutathione	None	Skin lightening
129.	Duperray et al., 2022	Randomized, double-blind, placebo- and benchmark-controlled clinical trial	Women	Oral L-cystine + reduced GSH	Placebo + benchmark	Skin pigmentation
130.	Richie et al., 2015	Randomized, double-blind, placebo-controlled clinical trial	Adults	Oral glutathione (250–1000 mg/day)	Placebo	Body GSH stores
131.	Weschawalit et al., 2017	Narrative review	N/A	GSH anti-aging & antimelanogenic effects	N/A	Mechanisms & evidence
132.	Sonthalia et al., 2016	Narrative review	N/A	GSH as whitening agent	N/A	Evidence & controversies
133.	Dilokthornsakul et al., 2019	Systematic review	N/A	Glutathione for skin color	N/A	Clinical outcomes
134.	Sarkar et al., 2025	Systematic review	N/A	GSH in melasma	N/A	Efficacy & safety

135.	Johnstone et al., 2018	Case series	7 patients	Contaminated glutathione infusions	None	Endotoxin poisoning
136.	Guilherme et al., 2017	Narrative review	N/A	Lipid nanoparticles for topical delivery	N/A	Formulation challenges
137.	Wu et al., 2024	In vivo + in vitro experimental	Mouse skin + cell lines	Liposome-thermo sensitive hydrogel	Standar d formulat ions	Anti-photoagi ng efficacy
138.	Patel et al., 2021	Narrative review	N/A	Lipid-based nanocarriers	N/A	Topical drug delivery
139.	Benson, 2017	Narrative review	N/A	Elastic liposomes	N/A	Transdermal delivery
140.	Carita et al., 2018	Narrative review	N/A	Liposomes for cutaneous delivery	N/A	Advances & perspectives
141.	Dinh et al., 2025	Narrative review	N/A	Hydrogel conjugation for drug delivery	N/A	Formulation engineering
142.	Gao et al., 2019	In vitro + in vivo experimental	Chronic wound models	Redox-responsive HA hydrogel	Standar d hydroge l	Wound healing, ROS response
143.	Li et al., 2024	In vivo + in vitro experimental	Complicate d wound models	Hydrogel-EV delivery system	Standar d care	Healing rate, inflammation
144.	Zhu et al., 2024	In vitro + in vivo experimental	Cell lines + mouse models	Acid & GSH dual-responsive hydrogel	Non-res ponsive hydroge l	Drug release, healing
145.	Liu et al., 2025	In vitro + ex vivo experimental	Skin models	SLN-enriched hydrogel with L-GSH	Standar d hydroge l	Anti-aging efficacy
146.	Santacroce et al., 2023	Narrative review	N/A	GSH in NAFLD	N/A	Pharmacologic al implications
147.	Dawi et al., 2025	Narrative review	N/A	GSH insufficiency in T2DM	N/A	Oxidative stress pathways
148.	Balendiran et al., 2004	Narrative review	N/A	GSH in cancer	N/A	Redox & tumor biology
149.	Desideri et al., 2019	Narrative review	N/A	Targeting GSH metabolism	N/A	Anticancer therapy
150.	Zhang et al., 2023	Narrative review	N/A	Cisplatin nephrotoxicity	N/A	Mechanisms & natural compou
151.	Casanova et al., 2020	Systematic review +	N/A	Protectants for cisplatin nephrotoxicity	N/A	Clinical efficacy

		meta-analysis				
152.	Yang et al., 2025	Narrative review	N/A	GSH-based metal nanomaterials	N/A	Functional applications
153.	Ramirez et al., 2024	Narrative review	N/A	Ethics in aesthetic medicine	N/A	Ethical frameworks
154.	da Prato et al., 2024	Narrative review	N/A	Patient-centered care ethics	N/A	Ethical foundations
155.	Waly et al., 2015	Human nutritional intervention	Healthy young adults	Low vitamin C diet	Normal diet	GSH depletion, oxidative stress
156.	Tram et al., 2021	In vitro experimental	Lens + retinal epithelial cells	GSH + vitamin C	Vitamin C alone	Antioxidant activity
157.	Gao et al., 2002	In vivo experimental	Myocardial reperfusion injury (rats)	GSH + ascorbic acid	GSH alone	Cardioprotection
158.	Guaiquil et al., 2001	In vitro experimental	HL-60 cells	Vitamin C in GSH-depleted cells	Untreated cells	Cell death inhibition
159.	Kapoor et al., 2025	Narrative review	N/A	Niacinamide for skin health	N/A	Dermatologic benefits
160.	Gehring, 2004	Narrative review	N/A	Niacinamide & skin	N/A	Mechanisms & effects
161.	Rolfe, 2014	Narrative review	N/A	Nicotinamide in skin diseases	N/A	Efficacy & side effects
162.	Serre et al., 2018	Narrative review	N/A	Regulation of melanogenesis	N/A	Pigmentation pathways
163.	Hakozaki et al., 2006	In vivo human cosmetic study	Healthy adults	Ultrasound + vitamin C + niacinamide	Vitamin C + niacinamide alone	Skin-lightening effect
164.	Foyer, 2001	Narrative review	N/A	Enhancing ascorbate & glutathione	N/A	Antioxidant prospects
165.	Colangelo et al., 2020	Systematic review	N/A	PDRN in wound healing	N/A	Regeneration outcome
166.	Jeong et al., 2017	In vivo experimental	Rat incisional wound model	PDRN treatment	Untreated wounds	Healing rate, collagen
167.	Khan et al., 2022	Narrative review	N/A	PDRN as anti-aging agent	N/A	Mechanisms & applications
168.	Park et al., 2022	In vitro experimental	Melanocyte models	Niacinamide + vitamin C + PDRN	Individual components	Melanogenesis markers

169.	Wahab et al., 2021	Randomized, double-blind, placebo-cont rolled clinical trial	Women	Oral + topical glutathione	Oral + topical placebo	Skin whitening
170.	Schmitt et al., 2015	Crossover clinical study	Healthy adults	NAC, oral GSH, sublingual GSH	Each other (crossov er)	Oxidative stress markers
171.	Yin et al., 2025	Narrative review	N/A	Oral GSH analogues	N/A	Bioavailability strategies
172.	Dehkordi & Ghasemi, 2024	Narrative review	N/A	GSH therapy challenges	N/A	Therapeutic strategies
173.	Shen & Wang, 2021	In vivo experimental	Diabetic rats	GSH liposomes	Untreate d diabetic rats	Oxidative stress, polyol pathway
174.	Xia et al., 2007	Narrative review	N/A	Lipid carriers for sunscreens	N/A	Formulation science
175.	Dobreva et al., 2020	Narrative review	N/A	Natural lipids in SLN/NLC	N/A	Topical delivery
176.	Villarama & Maibach, 2005	Narrative review	N/A	Glutathione as a depigmenting agent (oral, topical, intravenous; combinations with antioxidants)	N/A	Mechanisms of melanogenesis inhibition, tyrosinase modulation, melanin pathway shift, clinical depigmenting effects, safety considerations
177.	Estrela et al., 2006	Narrative review	N/A	GSH in cancer biology	N/A	Redox & therapy
178.	Marengo et al., 2020	Narrative review	N/A	miRNA regulation of GSH	N/A	Cancer progression
179.	Lusini et al., 2001	Observationa l biochemical study	Human renal carcinoma tissue	Measurement of GSH metabolism	Adjacen t normal tissue	GSH, GST, redox enzymes
180.	Lau et al., 2020	In vitro experimental	Colorectal cancer cells	High-glucose-ind uced GSH + rosiglitazone	Normal glucose	Apoptosis, ROS, chemosensitivi ty
181.	Bhowmick & Sarkar, 2020	Computation al mathematical modeling	In silico glioma models	ROS & GSH mathematical simulation	N/A	Tumor behavior modeling

182.	Theodossiou et al., 2017	In vitro experimental	Cancer cell lines	Hypericin PDT + GSH modulation	Normal GSH	PDT resistance, ROS
183.	De Luca et al., 2019	Structural biochemistry + in vitro	GSTP1-1 enzyme + cancer cells	Cisplatin + GSH interaction	Mutant GSTP1-1	Mechanism of resistance
184.	Alqarni et al., 2021	In vitro biochemical study	GSTA1-1 enzyme	Interaction with reactive dyes	No dye	Binding kinetics
185.	Hamid et al., 2022	Narrative review	N/A	Pharmacovigilance in primary care	N/A	Safety monitoring
186.	Song et al., 2023	Narrative review	N/A	Pharmacovigilance in China	N/A	System evolution
187.	Hadi et al., 2017	Narrative review	N/A	Pharmacists & ADR reporting	N/A	Reporting barriers
188.	Lorberbaum et al., 2015	Narrative review	N/A	Systems pharmacology & safety	N/A	Drug surveillance
189.	Jacob et al., 2013	Narrative review	N/A	Pharmacovigilance in ESRD	N/A	Safety challenges
190.	Alzahrani et al., 2025	Narrative review	N/A	GSH for skin lightening	N/A	Efficacy & safety
191.	Mackey & Liang, 2013	Narrative review	N/A	Counterfeit medicines	N/A	Global governance
192.	Jackson et al., 2012	Narrative review	N/A	Counterfeit medications (UK)	N/A	Public health impact
193.	Pathak et al., 2023	Narrative review	N/A	Tackling counterfeit drugs	N/A	Challenges & solutions
194.	Ozawa et al., 2018	Systematic review + meta-analysis	N/A	N/A	N/A	Prevalence, economic burden
