# Supplementary material for: Glutathione in Skin Aging and Tissue Regeneration: A Systematic Review of Molecular Mechanisms, Redox Modulation, and Biomedical Implications
Source: Molecules. 2026 Mar 15;31(6):981. doi: 10.3390/molecules31060981 (PMC13029213; doi:10.3390/molecules31060981)
Supplement: Supplementary file 1 [file molecules-31-00981-s001.zip › molecules-4169331 - Table S2 Risk of Bias.pdf]

Table S2. Risk of Bias Assessment According to the RoB 2 Tool

No	Study	Randomization Process	Deviations from Intended Interventions	Missing Outcome Data	Outcome Measurement	Selective Reporting	Overall Risk of Bias
2.	Guerrero-Navarro et al., 2024	Some concerns	Some concerns	Some concerns	Low	Low	Some concerns
8.	Kim et al., 2023	Some concerns	Some concerns	Some concerns	Low	Low	Some concerns
16.	Riskowski et al., 2019	Low	Low	Low	Low	Low	Low
18.	Cacciatore et al., 2010	Some concerns	Some concerns	Low	Low	Low	Some concerns
23.	Maestri et al., 2021	Some concerns	Some concerns	Low	Low	Low	Some concerns
25.	Dergousova et al., 2017	Low	Low	Low	Low	Low	Low
27.	Anashkina et al., 2023	Low	Low	Low	Low	Low	Low
29.	Kim et al., 2024	Low	Low	Low	Low	Low	Low
32.	Handy et al., 2009	Low	Low	Low	Low	Low	Low
33.	Sands et al., 2018	Some concerns	Low	Low	Low	Low	Some concerns
38.	Chełchowska et al., 2025	High	High	Low	Low	Low	High
41.	Hong et al., 2006	Some concerns	Some concerns	Low	Low	Low	Some concerns
49.	Schmidt et al., 2019	Some concerns	Some concerns	Low	Low	Low	Some concerns
54.	Pessoa et al., 2016	Some concerns	Some concerns	Low	Some concerns	Low	Some concerns
61.	Świderska-Kołacz et al., 2021	Low	Low	Low	Low	Low	Low
65.	Nogales et al., 2013	Low	Low	Low	Low	Low	Low
66.	Dogan et al., 2016	Low	Low	Low	Low	Low	Low
68.	Zhou et al., 2002	Low	Low	Low	Low	Low	Low
69.	Chen et al., 2021	Low	Low	Low	Low	Low	Low
73.	He et al., 2020	Some concerns	Some concerns	Low	Low	Low	Some concerns
76.	Kahl et al., 2018	Some concerns	Some concerns	Low	Low	Low	Some concerns
80.	Rozanski & Xu, 2002	Some concerns	Some concerns	Low	Some concerns	Low	Some concerns
84.	Cipollina et al., 2022	Low	Low	Low	Low	Low	Low
85.	Koike et al., 2007	Some concerns	Some concerns	Low	Low	Low	Some concerns

86.	Pekovic-Vaughan et al., 2014	Some concerns	Some concerns	Low	Low	Low	Some concerns
95.	Xiang et al., 2022	Low	Low	Low	Low	Low	Low
96.	Shang et al., 2016	Some concerns	Some concerns	Low	Low	Low	Some concerns
97.	Bonetti et al., 2024	Some concerns	Some concerns	Low	Some concerns	Low	Some concerns
104.	Chełchowska et al., 2025	Low	Low	Low	Low	Low	Low
109.	Tamas et al., 2021	Some concerns	Some concerns	Low	Low	Low	Some concerns
112.	Sekhar et al., 2022	Some concerns	Some concerns	Low	Low	Low	Some concerns
113.	Gronau et al., 2003	High	Some concerns	Low	Low	Low	High
124.	Watanabe et al., 2014	Low	Low	Low	Low	Low	Low
125.	Grandi et al., 2019	Low	Low	Low	Low	Low	Low
126.	Cui et al., 2024	Low	Low	Low	Low	Low	Low
127.	Arjinpathana & Asawanonda, 2012	Low	Low	Low	Low	Low	Low
128.	Handog et al., 2016	Some concerns	Low	Some concerns	Low	Low	Some concerns
129.	Duperray et al., 2022	Low	Low	Low	Low	Low	Low
130.	Richie et al., 2015	Low	Low	Low	Low	Low	Low
135.	Johnstone et al., 2018	High	High	Some concerns	Low	Low	High
137.	Wu et al., 2024	Some concerns	Some concerns	Low	Low	Low	Some concerns
142.	Gao et al., 2019	Some concerns	Some concerns	Low	Low	Low	Some concerns
143.	Li et al., 2024	Some concerns	Some concerns	Low	Low	Low	Some concerns
144.	Zhu et al., 2024	Some concerns	Some concerns	Low	Low	Low	Some concerns
145.	Liu et al., 2025	Low	Low	Low	Low	Low	Low
155.	Waly et al., 2015	Some concerns	Some concerns	Low	Some concerns	Low	Some concerns
156.	Tram et al., 2021	Low	Low	Low	Low	Low	Low
157.	Gao et al., 2002	Low	Low	Low	Low	Low	Low
158.	Guaiquil et al., 2001	Low	Low	Low	Low	Low	Low
163.	Hakozaki et al., 2006	High	Some concerns	Low	Some concerns	Low	High
166.	Jeong et al., 2017	Some concerns	Some concerns	Low	Low	Low	Some concerns
168.	Park et al., 2022	Low	Low	Low	Low	Low	Low

169.	Wahab et al., 2021	Low	Low	Low	Low	Low	Low
170.	Schmitt et al., 2015	Some concerns	Some concerns	Low	Low	Low	Some concerns
173.	Shen & Wang, 2021	Some concerns	Some concerns	Low	Low	Low	Some concerns
179.	Lusini et al., 2001	Some concerns	Some concerns	Low	Low	Low	Some concerns
180.	Lau et al., 2020	Low	Low	Low	Low	Low	Low
182.	Theodossiou et al., 2017	Low	Low	Low	Low	Low	Low
183.	De Luca et al., 2019	Low	Low	Low	Low	Low	Low
184.	Alqarni et al., 2021	Low	Low	Low	Low	Low	Low
